# Mitogenomic Profiling of *Cyclocheilichthys repasson* (Cypriniformes: Cyprinidae) and Its Phylogenetic Placement Within the Clade “Poropuntiinae”

**DOI:** 10.1002/ece3.71990

**Published:** 2025-08-13

**Authors:** Ayu Fitri Izaki, Sarifah Aini, Angkasa Putra, Hey‐Eun Kang, Ah Ran Kim, Soo Rin Lee, Mugi Mulyono, Muhammad Hilman Fu'adil Amin, Hyun‐Woo Kim, Shantanu Kundu

**Affiliations:** ^1^ Department of Marine Biology Pukyong National University Busan Republic of Korea; ^2^ Interdisciplinary Program of Marine and Fisheries Sciences and Convergent Technology Pukyong National University Busan Republic of Korea; ^3^ Institute of Marine Life Science Pukyong National University Busan Republic of Korea; ^4^ Institute of Marine Living Modified Organisms Pukyong National University Busan Republic of Korea; ^5^ Marine Integrated Biomedical Technology Center, National Key Research Institutes in Universities Pukyong National University Busan Republic of Korea; ^6^ Jakarta Technical University of Fisheries, Ministry of Marine Affairs and Fisheries Jakarta Republic of Indonesia; ^7^ Advance Tropical Biodiversity, Genomics, and Conservation Research Group, Department of Biology, Faculty of Science and Technology Airlangga University Surabaya Republic of Indonesia; ^8^ Research Center for Marine Integrated Bionics Technology Pukyong National University Busan Republic of Korea; ^9^ Department of Biology, Faculty of Science and Technology Airlangga University Surabaya Republic of Indonesia; ^10^ Ocean and Fisheries Development International Cooperation Institute, College of Fisheries Science Pukyong National University Busan Republic of Korea; ^11^ International Graduate Program of Fisheries Science Pukyong National University Busan Republic of Korea

**Keywords:** conservation, freshwater fish, matrilineal phylogeny, mitochondrial genome, southeast asia, Sundaland

## Abstract

The systematic status of the genus *Cyclocheilichthys* remains ambiguous due to a lack of comprehensive morphological and molecular evidence. Hence, this study represents the novel mitogenome of *Cyclocheilichthys repasson* to characterize its architecture and clarify its phylogenetic placement within the clade “Poropuntiinae.” The circular mitogenome was 16,571 base pairs in length and comprised 37 genes and a control region (CR). Most genes were encoded on the heavy strand, with the exception of *ND6* and eight tRNA genes located on the light strand. The genome exhibited an A + T bias of approximately 57.7% and displayed distinct AT‐ and GC‐skew patterns. All protein‐coding genes (PCGs) initiated with the standard ATG start codon, except for *COI*, which began with GTG. The amino acid composition was dominated by leucine, serine, threonine, and isoleucine, and the ratio of nonsynonymous to synonymous substitutions suggested strong purifying selection across all PCGs. The majority of transfer RNA genes exhibited the canonical cloverleaf secondary structure, with the exception of tRNA‐Ser, which lacked the dihydrouridine (D) stem. The comparative analysis of 24 “Poropuntiinae” species revealed four conserved sequence blocks within the CR, while tandem repeat motifs were exclusively detected in 11 species and absent in *C. repasson* . The mitogenome‐based phylogenetic analyses using Bayesian and Maximum Likelihood methods supported the monophyly of *Cyclocheilichthys* and sister relationship of *C. repasson* with *C. janthochir* and *C. apogon* . The partial *COI‐based* investigation further revealed potential cryptic diversity within *C. repasson* populations from both mainland (Laos) and island (Sumatra, Indonesia), as indicated by a genetic divergence of 1.06%. This population separation may have been influenced by historical paleo‐river networks across Southeast Asia/Sunda Shelf during periods of lower sea levels. Collectively, these findings offer valuable insights into the structural features of the mitochondrial genome and elucidate the evolutionary history among cyprinid fishes.

## Graphical abstract

In this study, we report the first complete mitochondrial genome of *Cyclocheilichthys repasson* from its native range in Indonesia. We characterize its genetic features in detail and elucidate its phylogenetic placement and matrilineal evolutionary history within the Clade “Poropuntiinae” in the family Cyprinidae. Additionally, we analyzed partial COI sequences from populations inhabiting both mainland and island ecosystems in Southeast Asia to infer historical dispersal patterns, potentially facilitated by ancient river networks.

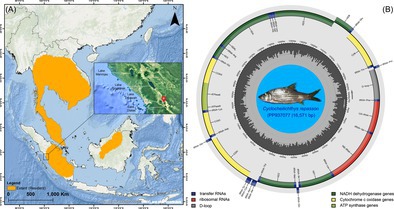

## 1 Introduction

The family Cyprinidae, belonging to the order Cypriniformes, represents the largest and most diverse group of fishes, comprising 1784 valid species classified into 166 genera (Mayden 1991; Fricke et al. 2025). This group plays a crucial role in the ecology and evolutionary dynamics of aquatic communities worldwide (Mayden 1991). Within the family Cyprinidae, the clade “Poropuntiinae” comprises approximately 100 species under 16 genera, representing a substantial component of the ichthyofaunal diversity (Khensuwan et al. 2023). The clade includes the genus *Cyclocheilichthys*, which comprises eight valid species ( *C. apogon* , *C. armatus* , *C. heteronema* , *C. janthochir* , *C. lagleri* , *C. repasson* , *C. schoppeae*, and *C. sinensis* ) inhabiting various freshwater ecosystems, like lakes, rivers, canals, ponds, and reservoirs (Rainboth 1996; Fricke et al. 2025). Most species within this genus are distributed across Southeast Asia, while *C. sinensis* is endemic to China (Fricke et al. 2025). The *Cyclocheilichthys* species possess considerable economic importance due to their utilization both as a food resource and in the ornamental fish trade (Kottelat et al. 1993; Rainboth 1996).

The taxonomy of *Cyclocheilichthys* has been a subject of scientific debate over the past decade, particularly regarding its monophyletic status. The morphological and phylogenetic analyses based on mitochondrial (*Cytb* and *COI*) and nuclear (*RAG1* and *RAG2*) markers initially suggested a division of the genus into two groups, with *Cyclocheilichthys* retained for *C. enoplos* and *Anematichthys* proposed for other species such as *A. apogon*, *A. armatus* , and *A. repasson*, thereby indicating the nonmonophyletic constitution of *Cyclocheilichthys* (Pasco‐Viel et al. 2012). However, subsequent studies revisited this classification and reinstated *C. apogon* , *C. armatus* , and *C. repasson* within the genus *Cyclocheilichthys*, while reassigning *C. enoplos* under the genus *Cyclocheilos* (Kottelat 2013; Pasco‐Viel et al. 2013). The latest taxonomic consensus retains *C. repasson* within *Cyclocheilichthys*, as reflected in Eschmeyer's Catalog of Fishes (Fricke et al. 2025). Ecologically, *C. repasson* is a benthopelagic species that feeds on aquatic insects and macrophytes (Rainboth 1996). This species is also regarded as a potamodromous cyprinid, inhabiting the middle to lower water columns in mainland and island regions of Southeast Asia (Froese and Pauly 2025).

Extensive research has been conducted to elucidate the biological, ecological, and taxonomic aspects of *Cyclocheilichthys* species. Studies on feeding ecology, seasonal dietary patterns, parasite diversity, autecology, epidemiology, and reproductive dynamics have been widely explored for *Cyclocheilichthys* (Nithiuthai et al. 2002; Hamid et al. 2015; Chavengkun et al. 2016; Rosli and Zain 2016; Nuraini et al. 2017; Juntaban et al. 2021). Furthermore, the systematic studies integrated both morphological and molecular data to provide insights into genetic diversity, phylogenetic relationships, population structure, and demographic history of *Cyclocheilichthys* species across Southeast Asia (Kenthao and Jearranaiprepame 2018; Kenthao et al. 2018; Roesma et al. 2023). With advancements in molecular approaches for biodiversity and systematics research, complete mitochondrial genome sequencing has emerged as a powerful tool for evolutionary assessments in fishes (Satoh et al. 2016). The utility of mitogenome analyses is driven by its maternal inheritance, highly conserved structure, and relatively high mutation rate, making it a valuable marker for evolutionary studies (Miya et al. 2003). The comparative mitogenome analyses are instrumental in investigating evolutionary diversification, ecological adaptations, phylogenetic relationships, and conservation implications, including those within cyprinid fishes (Hinsinger et al. 2015). To date, the mitogenomes of three *Cyclocheilichthys* species ( *C. apogon* , *C. heteronema* , and *C. janthochir* ) have been sequenced and are publicly available in GenBank (https://www.ncbi.nlm.nih.gov/nuccore). To further expand taxonomic coverage and enhance the understanding of the phylogenetic placement of “Poropuntiinae” species, this study aims to sequence the complete mitogenome of *C. repasson* from its native habitat in Indonesia. Additionally, we conduct comparative analyses of mitochondrial gene structures and their variations across *Cyclocheilichthys* species. The findings of this study are expected to contribute significantly to the taxonomic framework of *Cyclocheilichthys* and enhance our understanding of evolutionary relationships of the major lineage of the family Cyprinidae.

## 2 Materials and Methods

### 2.1 Sample Collection, Identification, and Preservation

**FIGURE 1:**
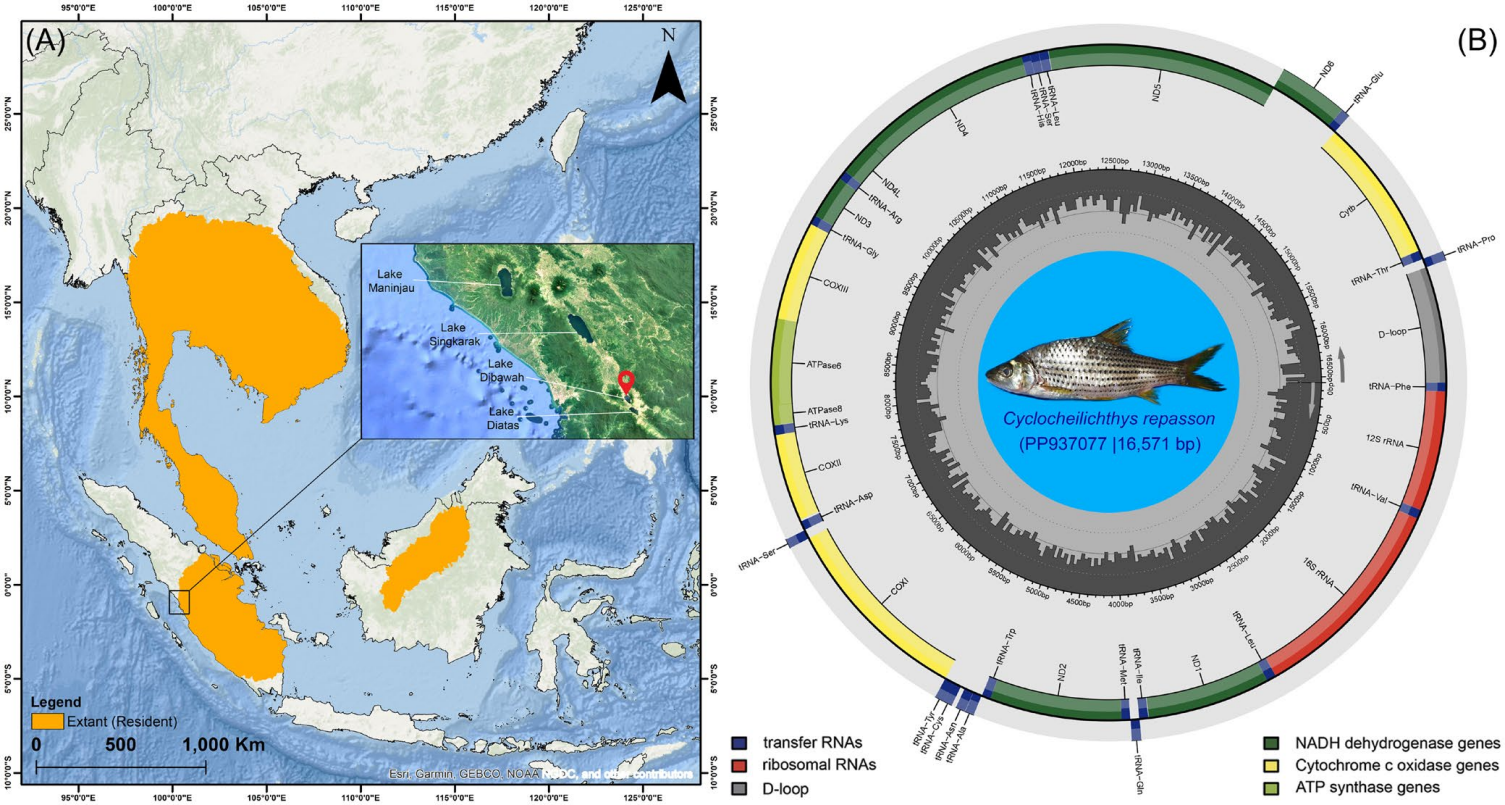
(A) The biogeographic distribution of *C. repasson* across mainland and island regions of Southeast Asia as per IUCN. The inset box highlights the specific locations of several major lakes in Sumatra. Specifically, the sampling site at Lake Dibawah is indicated by red pin. (B) The circular representation of the generated mitogenome of *C. repasson* was annotated using the MitoAnnotator tool. The colored arcs represent the positions of various mitochondrial genes, including PCGs, rRNAs, tRNAs, and CR. The species photograph was provided by Mrs. Yossi Agusta (Government of Solok Regency, West Sumatra Province, Republic of Indonesia).

A single cyprinid fish specimen was collected from Lake Dibawah, Sumatra, Indonesia (1.026389 S 100.739722 E). The species identification was confirmed based on the morphological characteristics of *C. repasson* described in previous studies (Roberts 1989; Pasco‐Viel et al. 2012). Notably, the diagnostic features of this species include a series of spots along the lateral scale rows, a distinct black blotch at the base of the caudal fin, two pairs of barbels surrounding the mouth serving as sensory organs, and a maximum standard length of up to 28 cm. The collected specimen was euthanized by adding 2‐phenoxyethanol directly into the aquarium at a concentration of 600 μL L^−1^ (Nahon et al. 2017). Upon confirmation of death, the specimen was rinsed three times with Milli‐Q water (Merck‐Millipore, Molsheim, France) to prepare for molecular analysis. Under aseptic conditions, approximately 20 g of dorsal muscle tissue was carefully excised parallel to the lateral line. The tissue sample was immediately placed into 2 mL centrifuge tubes containing 95% molecular‐grade ethanol and stored at −20°C to prevent DNA degradation and microbial contamination. The specimen was preserved as a voucher under the code “IDN7” and deposited at the Jakarta Technical University of Fisheries, Pariaman Campus, Ministry of Marine Affairs and Fisheries, Indonesia. The subsequent molecular analyses were conducted at the Molecular Physiology Laboratory, Pukyong National University, Busan, South Korea. Additionally, the distribution data of *C. repasson* were mapped from the IUCN Red List of Threatened Species database to evaluate its biogeographical patterns in Southeast Asia (Figure 1A).

### 2.2 Ethics Statement

The focal species ( *C. repasson* ) is locally consumed as a small food fish along with other cyprinids and is currently classified as “Least Concern” on the IUCN Red List of Threatened Species (https://www.iucnredlist.org/). Therefore, no specific permissions were required for its collection. However, all procedures were conducted in compliance with the relevant institutional guidelines and regulations approved by the host institution (Approval No. PKNUIACUC‐2025‐16). Furthermore, the experimental protocols adhered to the ARRIVE 2.0 guidelines (https://arriveguidelines.org) (Percie du Sert et al. 2020).

### 2.3 Genomic DNA Extraction and *COI‐Based* Validation

The genomic DNA was isolated from approximately 30 mg of muscle tissue of *C. repasson* using the AccuPrep Genomic DNA Extraction Kit (Bioneer, Daejeon, South Korea), following the manufacturer's standard protocol. Tissue homogenization was performed in 600 μL of 1× lysis buffer using a TissueLyser II system (Qiagen, Hilden, Germany) for 60 s. To promote efficient cell lysis and protein degradation, the homogenate was treated with 100 μL of sodium dodecyl sulfate (SDS) and 20 μL of proteinase K, followed by incubation at 60°C for 12 h. The genomic DNA was precipitated by the addition of 500 μL of GC buffer and 300 μL of isopropanol, then transferred to a spin column and centrifuged at 7,155 *g* for 1 min. The bound DNA was washed sequentially with Wash Buffers 1 and 2 to eliminate impurities and eluted in 50 μL of TE buffer for subsequent applications. The DNA concentration and purity were assessed using a NanoDrop spectrophotometer (Thermo Fisher Scientific, D1000, Waltham, MA, USA). To amplify the partial mitochondrial *COI* gene, the universal primers Fish‐BCH (5′‐TCAACYAATCAYAAAGATATYGGCAC‐3′) and Fish‐BCL (5′‐ACTTCYGGGTGRCCRAARAATCA‐3′) were used (Baldwin et al. 2009). The polymerase chain reaction (PCR) was carried out in a 30 μL reaction volume consisting of 1 μL of each primer, 0.9 μL of 3% dimethyl sulfoxide (DMSO), 19.9 μL of nuclease‐free water, 3 μL of 10× ExTaq Buffer, 0.2 μL of Ex Taq Hot Start DNA polymerase, 3 μL of dNTPs, and 1 μL of a 1:10 diluted genomic DNA template. The PCR cycling conditions were as follows: initial denaturation at 94°C for 3 min; 40 cycles of denaturation at 94°C for 30 s, annealing at 50°C for 30 s, and extension at 72°C for 1 min; followed by a final extension at 72°C for 5 min. The amplified PCR products were purified using the AccuPrep PCR/Gel Purification Kit (Bioneer, South Korea) and subjected to bidirectional sequencing using an ABI PRISM 3730XL DNA Analyzer (Macrogen, Daejeon, South Korea). The raw sequence data were inspected and edited using SeqScanner version 1.0 (Applied Biosystems Inc., Foster City, CA, USA) to remove ambiguous or low‐quality regions.

### 2.4 Mitogenome Sequencing and Assembly

To generate the mitogenome of *C. repasson* , paired‐end sequencing (2 × 150 bp) was conducted using the NovaSeq platform (Illumina, San Diego, CA, USA) at Macrogen (Daejeon, South Korea; https://dna.macrogen.com/). The genomic DNA (100 ng) was sheared into fragments of appropriate length using a Covaris adaptive focused acoustic system (Covaris, Woburn, MA, USA), generating blunt‐ended, double‐stranded DNA with 5′ phosphorylation. The library construction was carried out using the TruSeq Nano DNA High‐Throughput Library Prep Kit (Illumina), following the manufacturer's protocol. The fragmented DNA underwent end‐repair, bead‐based size selection, 3′‐adenylation, and ligation with TruSeq DNA UD Indexing adapters. The resulting library was then enriched via PCR amplification. Quantification of the final DNA library was performed using quantitative PCR with the KAPA Library Quantification Kit, and quality assessment was carried out using the Agilent 4200 TapeStation system with D1000 ScreenTape (Agilent Technologies, Santa Clara, CA, USA). Further, high‐quality raw reads obtained from sequencing were assembled using Geneious Prime version 2023.0.1, with mapping guided by the mitogenome of a closely related species, *Cyclocheilos enoplos* (Accession No. AP011371) (Kearse et al. 2012). The sequence segments refinement and alignment of overlapping regions were performed using MEGA version 12 to ensure sequence continuity and accuracy (Kumar et al. 2024). Annotation of gene boundaries, coding regions, and gene orientations was conducted using both the MITOS web server (http://mitos.bioinf.uni‐leipzig.de) and MitoAnnotator (http://mitofish.aori.u‐tokyo.ac.jp/annotation/input/) (Bernt et al. 2013; Iwasaki et al. 2013). The protein‐coding genes (PCGs) were further validated using the Open Reading Frame Finder (ORFfinder; https://www.ncbi.nlm.nih.gov/orffinder/) by translating nucleotide sequences into amino acid sequences to confirm reading frames and coding integrity. The finalized mitogenome sequence of *C. repasson* was deposited in the GenBank database, where it was assigned a specific accession number.

### 2.5 Mitogenome Characterization and Comparative Analyses

In this study, a circular map of the mitogenome of *C. repasson* was generated using MitoAnnotator to aid in the visualization of its genomic architecture. The structural organization of the mitogenome was examined and compared with those of three closely related congeners: *C. janthochir* (Accession No. AP011185), *C. apogon* (AP011250), and *C. heteronema* (AP011380). The intergenic spacers and gene overlaps were manually identified using Microsoft Excel version 16. The start and stop codons for PCGs were annotated using a combination of MEGA version 12 and MITOS outputs. The nucleotide composition analyses were performed for the 13 PCGs, two ribosomal RNA (rRNA) genes, 22 transfer RNA (tRNA) genes, and the control region (CR) using MEGA version 12. The AT‐skew and GC‐skew values were calculated using the following formulas: AT‐skew = (A − T)/(A + T) and GC‐skew = (G − C)/(G + C), respectively (Perna and Kocher 1995). The nucleotide diversity (π) across the mitogenome of *Cyclocheilichthys* was determined using a sliding window method implemented in DnaSP version 6.0, with a window length of 200 base pairs (bp) and a step size of 25 bp (Rozas et al. 2017). The codon saturation in PCGs was assessed using DAMBE version 6 based on the relative rates of transitions and transversions (Xia 2017). Further analyses included the assessment of relative synonymous codon usage (RSCU), amino acid composition, and pairwise comparisons of synonymous (Ks) and nonsynonymous (Ka) substitution rates between *C. repasson* and its congeneric counterparts using DnaSP version 6.0. The boundaries of rRNA and tRNA genes were verified using the tRNAscan‐SE Search Server version 2.0 and ARWEN version 1.2 (Laslett and Canbäck 2008; Chan et al. 2021). The putative structural domains within the CR were delineated through multiple sequence alignments of the “Poropuntiinae” clade, performed using CLUSTAL X (Thompson et al. 1997; Satoh et al. 2016). To detect potential repeat motifs within the CR, we utilized the Tandem Repeats Finder algorithm (https://tandem.bu.edu/trf/trf.html), given the importance of repeats in developing genetic markers for population structure and evolutionary investigations (Benson 1999). Although the dataset in this study comprises 29 species from the “Poropuntiinae” clade, only 24 species possess an available CR sequence. The remaining five species were excluded from the analysis due to the absence of CR sequences, including *Eirmotus octozona* (AP011367), *Poropuntius huangchuchieni* (MN723896), *Poropuntius hampaloides* (AP011312), *Puntioplites falcifer* (AP011248), and *Puntioplites waandersi* (AP011249).

### 2.6 Mitogenome‐Based Phylogenetic Assessment

To elucidate the phylogenetic position of *C. repasson* within the broader Cyprinidae lineage, the complete mitogenome sequences from 39 valid species were compiled from the GenBank database. This dataset included 29 representative species (one generated and 28 database) from the “Poropuntiinae” clade. In addition, the dataset encompassed one species of each subfamily (Labeoninae, Cyprininae, Acrossocheilinae, Barbinae, Probarbinae, Schizopygopsinae, Schizothoracinae, Smiliogastrinae, Spinibarbinae, and Torinae), as well as one species ( *Chagunius chagunio* ) classified under Cyprinidae *incertae sedis* (Table S1). Overall, the “Poropuntiinae” clade analyzed represents approximately 26.61% of the taxonomically valid species currently listed in Eschmeyer's Catalog of Fishes (Fricke et al. 2025). The mitogenome of the *Rasbora dusonensis* (Accession No. MW232454), a member of the family Danionidae, was designated as the outgroup. The phylogenetic relationships were reconstructed using both Bayesian (BA) and Maximum Likelihood (ML) approaches. A concatenated dataset of 13 PCGs was assembled using iTaxoTools version 0.1 (Vences et al. 2021). The best‐fit nucleotide substitution model, identified as GTR + G + I, was selected based on the Bayesian Information Criterion (BIC) using PartitionFinder version 2 and JModelTest version 2 (Darriba et al. 2012; Miller et al. 2015; Lanfear et al. 2017). The BA analysis was performed using MrBayes version 3.1.2 with the Metropolis‐coupled Markov Chain Monte Carlo (MCMC) algorithm, applying the nst = 6 setting. The chains were run for 10,000,000 generations with sampling every 100 generations, and the first 25% of trees were discarded as burn‐in (Ronquist et al. 2012). The ML tree was inferred using PhyML version 3.0 with the same substitution model (Guindon et al. 2010; Trifinopoulos et al. 2016). All resulting phylogenetic trees were visualized using the Interactive Tree of Life (iTOL) web server version 6 to enhance clarity and presentation (Letunic and Bork 2024).

### 2.7 *COI* ‐Based Genetic Distance and Phylogeny

To assess the genetic diversity and demographic distinctiveness of *C. repasson* , an additional dataset comprising partial sequences of the mitochondrial *COI* gene (582 bp) was assembled. This dataset included newly generated sequences (Accession Nos. mitogenome: PP937077 and partial *COI*: PX022682) and a previously published sequence from mainland Laos (JQ346165) (Pasco‐Viel et al. 2012). To enhance the comparative framework, 10 *COI* sequences of two congeners, *C. janthochir* and *C. apogon* , were also retrieved from GenBank (Table S2). The genetic distances were calculated using the Kimura 2‐parameter (K2P) model implemented in MEGA version 12. Both BA and ML phylogenetic analyses were conducted based on this *COI* dataset, employing *Labiobarbus lineatus* (AP012153) as an outgroup. The BA analysis was performed using MrBayes version 3.1.2, while the ML tree was inferred using PhyML version 3.0. Both phylogenetic trees were visualized using the iTOL web server version 6.

## 3 Results

### 3.1 Mitogenome Organization and Structure

The generated *COI* sequence was validated using nucleotide BLAST (https://blast.ncbi.nlm.nih.gov), showing 98.97% identity with a reference sequence from the GenBank database (Accession No. JQ346165), thereby confirming species identity. The mitogenome of *C. repasson* was successfully assembled and annotated, and it revealed a typical circular structure with a total length of approximately 16,571 bp. The mitogenome was deposited in GenBank under the accession number PP937077 (Figure 1B). It consisted of 13 PCGs, 22 tRNA genes, two rRNA genes, and one noncoding CR, consistent with the typical mitogenome organization found in vertebrates. The comparative analysis of the four *Cyclocheilichthys* species examined in this study dataset showed that mitogenome sizes ranged from 16,571 bp in *C. repasson* to 16,586 bp in *C. apogon* , and all mitogenomes exhibited conserved vertebrate mitochondrial architecture. In *C. repasson* , most mitochondrial genes were encoded on the heavy strand (H‐strand), except for *ND6* and eight tRNA genes (*tRNA‐Gln*, *tRNA‐Ala*, *tRNA‐Asn*, *tRNA‐Cys*, *tRNA‐Tyr*, *tRNA‐Ser*, *tRNA‐Glu*, and *tRNA‐Pro*), which were located on the light strand (L‐strand) (Table 1). The nucleotide composition of the *C. repasson* mitogenome was 33.04% adenine (A), 24.66% thymine (T), 15.20% guanine (G), and 27.10% cytosine (C), resulting in an A + T content of 57.70%. The AT‐skew and GC‐skew values were calculated as 0.145 and −0.281, respectively. Among the *Cyclocheilichthys* mitogenomes analyzed, the A + T content ranged from 57.70% in *C. repasson* to 58.54% in *C. heteronema* . The AT‐skew values also ranged from 0.116 in *C. heteronema* to 0.155 in *C. janthochir* , while GC‐skew values ranged from −0.288 in *C. janthochir* to −0.262 in *C. heteronema* (Table 2).

**TABLE 1:** Gene organization of the complete mitochondrial genome of *C. repasson* , detailing gene positions, strand orientation, gene lengths, and intergenic nucleotides. The letters “H” and “L” indicate genes encoded on the heavy and light strands, respectively, while “–” denotes an incomplete stop codon.

Genes	Start	End	Strand	Size (bp)	Intergenic nucleotide	Anti‐codon	Start codon	Stop codon
*tRNA‐Phe*	1	69	H	69	0	GAA	˙	˙
*12S rRNA*	70	1022	H	953	0	˙	˙	˙
*tRNA‐Val*	1023	1094	H	72	0	TAC	˙	˙
*16S rRNA*	1095	2775	H	1681	0	˙	˙	˙
*tRNA‐Leu*	2776	2851	H	76	0	TAA	˙	˙
*ND1*	2852	3826	H	975	4	˙	ATG	TAA
*tRNA‐Ile*	3831	3902	H	72	−2	GAT	˙	˙
*tRNA‐Gln*	3901	3971	L	71	1	TTG	˙	˙
*tRNA‐Met*	3973	4041	H	69	0	CAT	˙	˙
*ND2*	4042	5086	H	1045	0	˙	ATG	T‐‐
*tRNA‐Trp*	5087	5157	H	71	2	TCA	˙	˙
*tRNA‐Ala*	5160	5228	L	69	1	TGC	˙	˙
*tRNA‐Asn*	5230	5302	L	73	33	GTT	˙	˙
*tRNA‐Cys*	5336	5402	L	67	−1	GCA	˙	˙
*tRNA‐Tyr*	5402	5472	L	71	1	GTA	˙	˙
*COI*	5474	7021	H	1548	−1	˙	GTG	TAA
*tRNA‐Ser*	7021	7092	L	72	3	TGA	˙	˙
*tRNA‐Asp*	7096	7167	H	72	14	GTC	˙	˙
*COII*	7182	7872	H	691	0	˙	ATG	T‐‐
*tRNA‐Lys*	7873	7948	H	76	1	TTT	˙	˙
*ATP8*	7950	8114	H	165	**−7**	˙	ATG	TAG
*ATP6*	8108	8790	H	683	0	˙	ATG	TA‐
*COIII*	8791	9575	H	785	0	˙	ATG	TA‐
*tRNA‐Gly*	9576	9648	H	73	0	TCC	˙	˙
*ND3*	9649	9997	H	349	0	˙	ATG	T‐‐
*tRNA‐Arg*	9998	10,067	H	70	0	TCG	˙	˙
*ND4L*	10,068	10,364	H	297	−7	.	ATG	TAA
*ND4*	10,358	11,738	H	1381	0	.	ATG	T‐‐
*tRNA‐His*	11,739	11,807	H	69	0	GTG	˙	˙
*tRNA‐Ser*	11,808	11,877	H	70	1	GCT	˙	˙
*tRNA‐Leu*	11,879	11,951	H	73	3	TAG	˙	˙
*ND5*	11,955	13,778	H	1824	−4	˙	ATG	TAA
*ND6*	13,775	14,296	L	522	0	˙	ATG	TTA
*tRNA‐Glu*	14,297	14,365	L	69	5	TTC	˙	˙
*Cytb*	14,371	15,511	H	1141	0	˙	ATG	TT‐
*tRNA‐Thr*	15,512	15,583	H	72	−1	TGT	˙	˙
*tRNA‐Pro*	15,583	15,653	L	71	0	TGG	˙	˙
Control region	15,654	16,571	H	918	˙	˙	˙	˙

**TABLE 2:** Size and nucleotide composition of the complete mitochondrial genomes and different genes across various *Cyclocheilichthys* species, including the proportions of adenine (A), thymine (T), guanine (G), and cytosine (C), as well as the overall A + T and G + C content.

Species name	Size (bp)	A%	T%	G%	C%	A + T%	AT‐Skew	GC‐Skew
**Complete mitogenome**								
*C. repasson*	16,571	33.04	24.66	15.20	27.10	57.70	0.145	−0.281
*C. janthochir*	16,580	33.66	24.64	14.86	26.85	58.30	0.155	−0.288
*C. apogon*	16,586	33.37	24.87	14.89	26.87	58.24	0.146	−0.287
*C. heteronema*	16,573	32.66	25.88	15.30	26.16	58.54	0.116	−0.262
**PCGs**								
*C. repasson*	11,406	31.15	26.64	14.60	27.62	57.79	0.078	−0.308
*C. janthochir*	11,409	31.94	26.71	14.16	27.20	58.65	0.089	−0.315
*C. apogon*	11,408	31.74	27.05	14.07	27.14	58.79	0.080	−0.317
*C. heteronema*	11,415	30.70	27.74	14.88	26.69	58.43	0.051	−0.284
**rRNAs**								
*C. repasson*	2587	35.21	19.64	20.49	24.66	54.85	0.284	−0.092
*C. janthochir*	2636	35.62	19.20	20.30	24.89	54.82	0.300	−0.102
*C. apogon*	2634	35.23	19.17	20.58	25.02	54.40	0.295	−0.097
*C. heteronema*	2634	35.65	20.12	20.05	24.18	55.77	0.278	−0.094
**tRNAs**								
*C. repasson*	1567	31.14	24.44	19.40	25.02	55.58	0.121	−0.126
*C. janthochir*	1563	28.92	27.38	23.10	20.60	56.30	0.027	0.057
*C. apogon*	1564	28.96	27.17	23.15	20.72	56.14	0.032	0.055
*C. heteronema*	1568	29.15	28.64	22.64	19.58	57.78	0.009	0.073
**CRs**								
*C. repasson*	918	36.17	32.57	12.09	19.17	68.74	0.052	−0.226
*C. janthochir*	926	35.21	32.18	12.63	19.98	67.39	0.045	−0.225
*C. apogon*	932	34.76	31.33	12.45	21.46	66.09	0.052	−0.266
*C. heteronema*	917	34.68	33.91	13.09	18.32	68.59	0.011	−0.167

### 3.2 Intergenic Spacer and Overlapping Regions

In this study, the mitogenome of *C. repasson* was characterized by the presence of 11 intergenic spacer regions and seven overlapping regions between adjacent genes (Table S3). The longest intergenic spacer, measuring 33 bp, was located between *tRNA‐Asn* and *tRNA‐Cys*. Additional significant intergenic spacers included a 14 bp region between *tRNA‐Asp* and *COII*, a 5 bp spacer between *tRNA‐Glu* and *Cytb*, and a 4 bp spacer between *ND1* and *tRNA‐Ile*. Moreover, multiple shorter intergenic spacers ranging from 1 to 3 bp were dispersed throughout the mitogenome. The gene overlaps were most prominent between *ATP8* and *ATP6* (7 bp), *ND4L* and *ND4* (7 bp), and *ND5* and *ND6* (4 bp). The shorter overlaps of 1–2 bp were also observed at several loci, including *tRNA‐Ile* and *tRNA‐Gln* (2 bp), *tRNA‐Cys* and *tRNA‐Tyr* (1 bp), *COI* and *tRNA‐Ser* (S2) (1 bp), and *tRNA‐Thr* and *tRNA‐Pro* (1 bp). The comparative analysis with three congeneric species ( *C. janthochir* , *C. apogon* , and *C. heteronema* ) indicated that the overall arrangement of intergenic spacers and overlapping regions in *C. repasson* was largely conserved. The longest intergenic spacer between *tRNA‐Asn* and *tRNA‐Cys* was conserved at 33 bp in *C. janthochir* and *C. apogon* , but was slightly shorter in *C. heteronema* (32 bp). The intergenic spacer between *tRNA‐Asp* and *COII* was 13 bp in both *C. janthochir* and *C. apogon* , compared to 16 bp in *C. heteronema* . A significant variation was observed at the junction between *COI* and *tRNA‐Ser* (S2), where *C. repasson* exhibited a 1 bp gene overlap, whereas *C. janthochir* and *C. apogon* displayed no overlap, and *C. heteronema* showed a longer overlap of 8 bp. Additionally, the intergenic spacer between *tRNA‐Leu* (L1) and *ND5* varied across species, ranging from 2 bp in *C. heteronema* , 3 bp in *C. repasson* , and 4 bp in both *C. janthochir* and *C. apogon* (Table S3).

### 3.3 Protein‐Coding Genes Features

The mitogenome of *C. repasson* comprises 13 PCGs with a total length of 11,406 bp, accounting for approximately 68.85% of the entire sequence. The nucleotide composition of the PCGs showed a pronounced A + T bias (57.79%), with AT‐skew and GC‐skew values calculated as 0.078 and −0.308, respectively. In comparison with three congeneric species— *C. janthochir* , *C. apogon* , and *C. heteronema* —the *C. repasson* exhibited the shortest total PCG length. The PCG lengths among these species ranged from 11,406 bp ( *C. repasson* ) to 11,415 bp ( *C. heteronema* ). The A + T bias of the congeners was slightly higher, ranging from 58.43% in *C. heteronema* to 58.79% in *C. apogon* . Similarly, AT‐skew values varied from 0.051 ( *C. heteronema* ) to 0.089 ( *C. janthochir* ), while GC‐skew values ranged from −0.317 in *C. apogon* to −0.284 in *C. heteronema* (Table 2). In *C. repasson* , all PCGs initiated with the standard ATG start codon, except for *COI*, which began with GTG—a pattern also observed in *C. janthochir* , *C. apogon* , and *C. heteronema* (Table S4). However, the termination codons exhibited greater variability. Notably, six genes in *C. repasson* (*ND1*, *COI*, *ATP8*, *ND4L*, *ND5*, and *ND6*) terminated with complete stop codons (TAA or TAG), whereas the remaining genes ended with incomplete termination signals: a single thymine (T‐‐) in *ND2*, *COII*, *ND3*, and *ND4*, or a two‐nucleotide codon (TA‐) in *ATP6* and *COIII*. The *Cytb* gene concluded with TT‐, presumed to represent an incomplete stop codon. In addition, the *COI* gene in *C. heteronema* terminated with the atypical AGG codon, contrasting with the TAA stop codon found in the other three species. The similar patterns of incomplete termination codons were observed across the congeners, suggesting the likely involvement of posttranscriptional polyadenylation in completing these truncated stop signals. Nevertheless, the subtle interspecific differences were evident. Specifically, the *COIII* ended with TA‐ in *C. repasson* , *C. janthochir* , and *C. heteronema* , but terminated with T‐‐ in *C. apogon* . Furthermore, the *ND4* gene terminated with TT‐ exclusively in *C. heteronema* , while the other species exhibited a T‐‐ termination (Table S4).

### 3.4 Substitutions Pattern and Relative Synonymous Codon Usage

**FIGURE 2:**
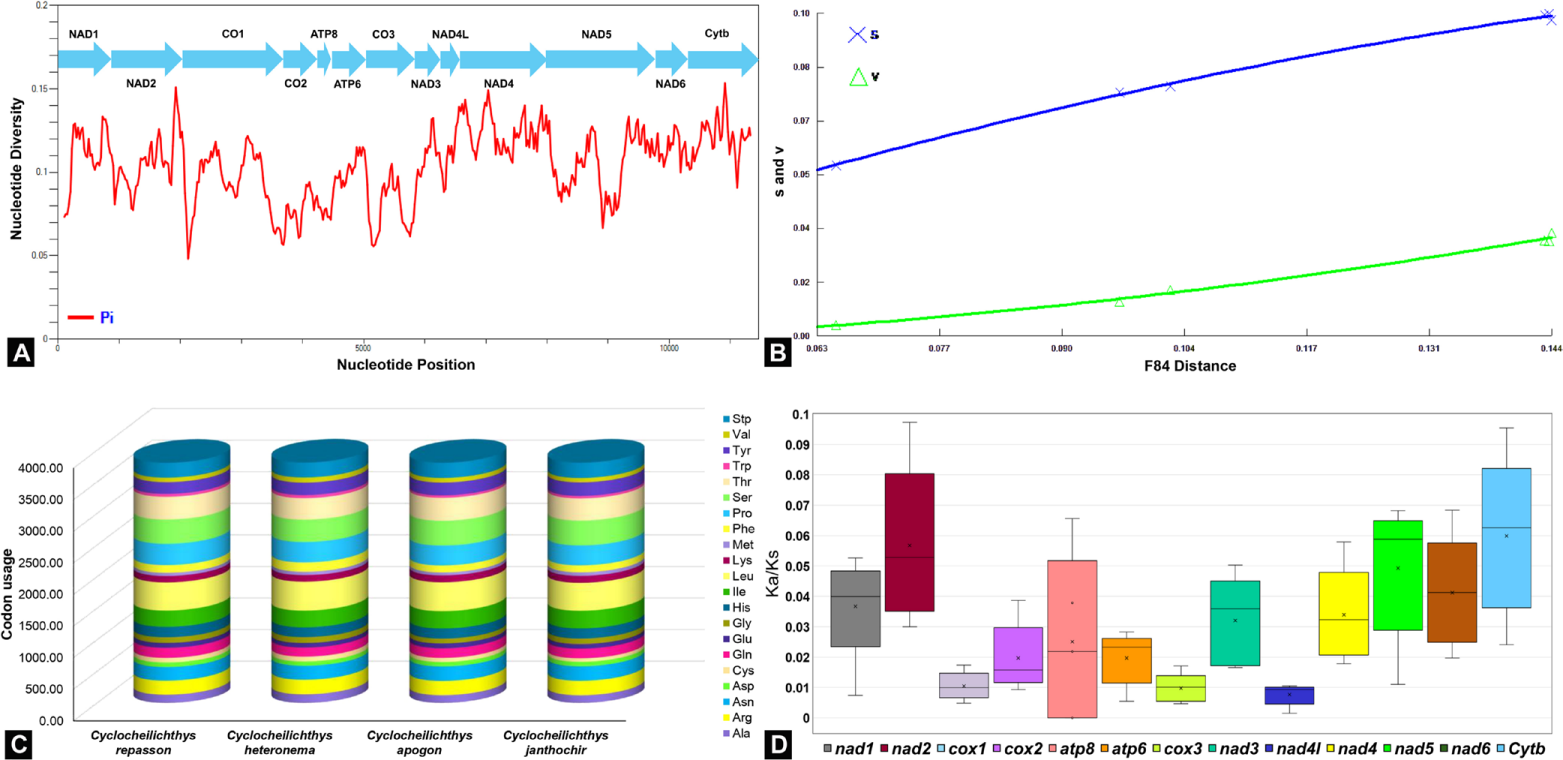
(A) Nucleotide diversity (π) across mitochondrial PCGs among *Cyclocheilichthys* species, (B) Scatter plot showing the relationship between transitions (s) and transversions (v) with genetic divergence in PCGs, based on F84 substitution distances, (C) Codon usage abundance in the mitogenomes of four different *Cyclocheilichthys* species. The color code for each of the 20 standard amino acids is shown to the right of the plot, and (D) Boxplot illustrating pairwise Ka/Ks ratios for each mitochondrial PCG among *Cyclocheilichthys* species, including *C. repasson* .

**FIGURE 3:**
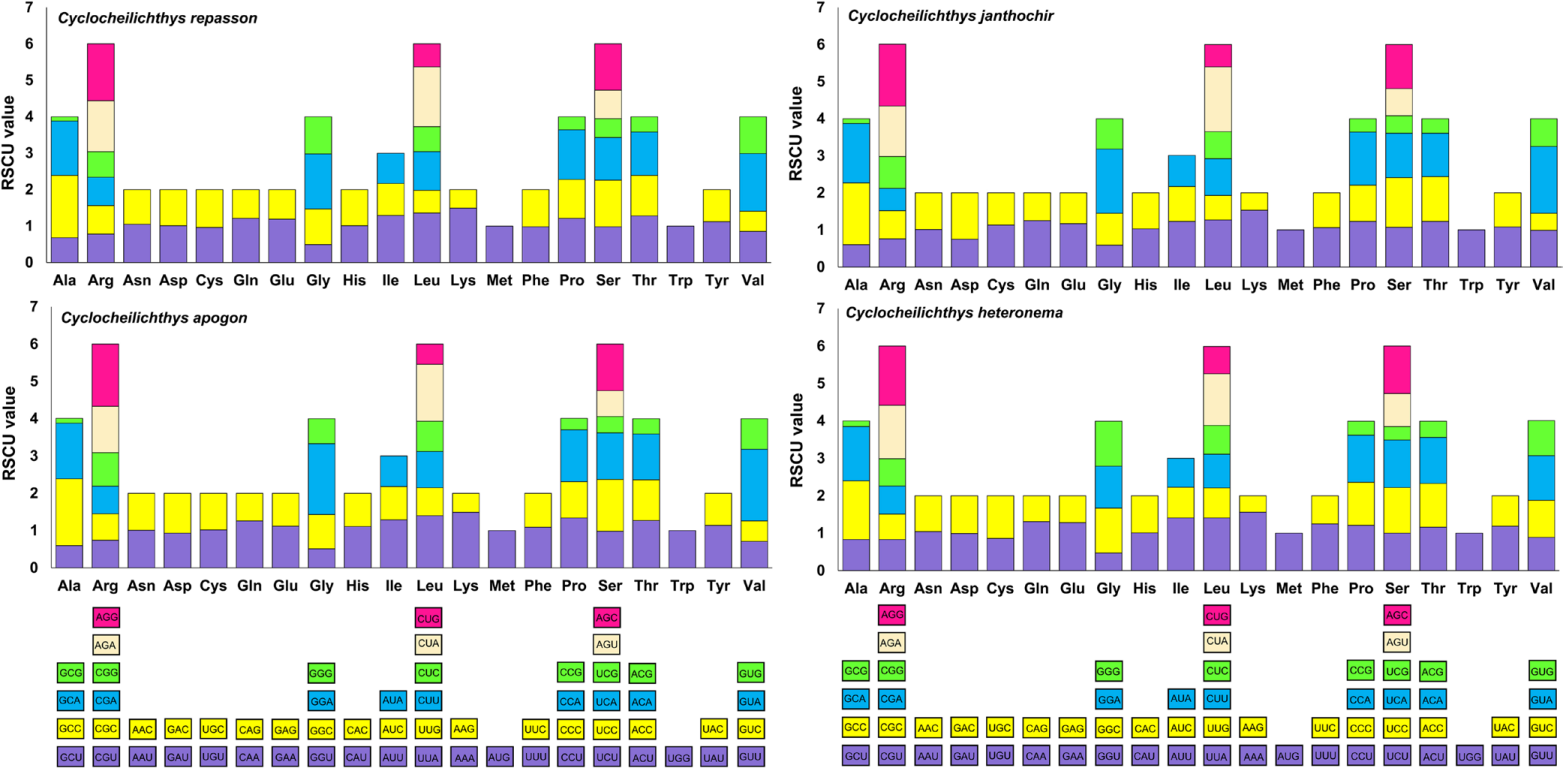
The comparative analysis of relative synonymous codon usage (RSCU) among four *Cyclocheilichthys* species. The collective RSCU values are plotted on the *y*‐axis against codons corresponding to each amino acid on the *x*‐axis.

In‐depth analysis, the nucleotide diversity (π) was assessed, and the highest π value was found in the base region 10,811–11,010 of the *Cytb* gene (π = 0.153), indicating the greatest level of polymorphism among the PCGs, followed by the *ND2* gene with a π value of 0.140. Conversely, the *COI* gene exhibited the lowest π value of 0.048, suggesting it was the most conserved gene among the analyzed PCGs (Figure 2A). The saturation analysis revealed that neither transitions nor transversions experienced saturation, as the F84 divergence values consistently increased across all PCGs of the *Cyclocheilichthys* mitogenomes (Figure 2B). The four species examined showed similar amino acid abundances, reflecting comparable codon usage preferences among them. Leucine (Leu), serine (Ser), threonine (Thr), and isoleucine (Ile) were the most abundant amino acids across all species. Notably, the amino acid composition of *C. repasson* consisted of 6.79% Ile, 9.47% Thr, 10.11% Ser, and 11.74% Leu, while methionine (Met), valine (Val), and tryptophan (Trp) were the least abundant amino acids (Figure 2C). To evaluate selective pressures acting on the PCGs of *C. repasson* and its congeneric, the ratio of Ka/Ks was calculated for each gene. All 13 PCGs exhibited *K*_a_/*K*_s_ values below “1,” with pairwise average ratios ranging from 0.0093 ± 0.0012 for *ND4L* to 0.0687 ± 0.0240 for *Cytb* (Figure 2D; Table S5). The codon usage analysis showed that the GCC codon, coding for alanine (Ala), had the highest frequency across all *Cyclocheilichthys* species, with an average RSCU value of 1.69. Meanwhile, the AUG codon for methionine (Met) and the UGG codon for tryptophan (Trp) both exhibited an RSCU value of 1 across all analyzed species (Figure 3; Table S6).

### 3.5 Ribosomal and Transfer RNA Genes

**FIGURE 4:**
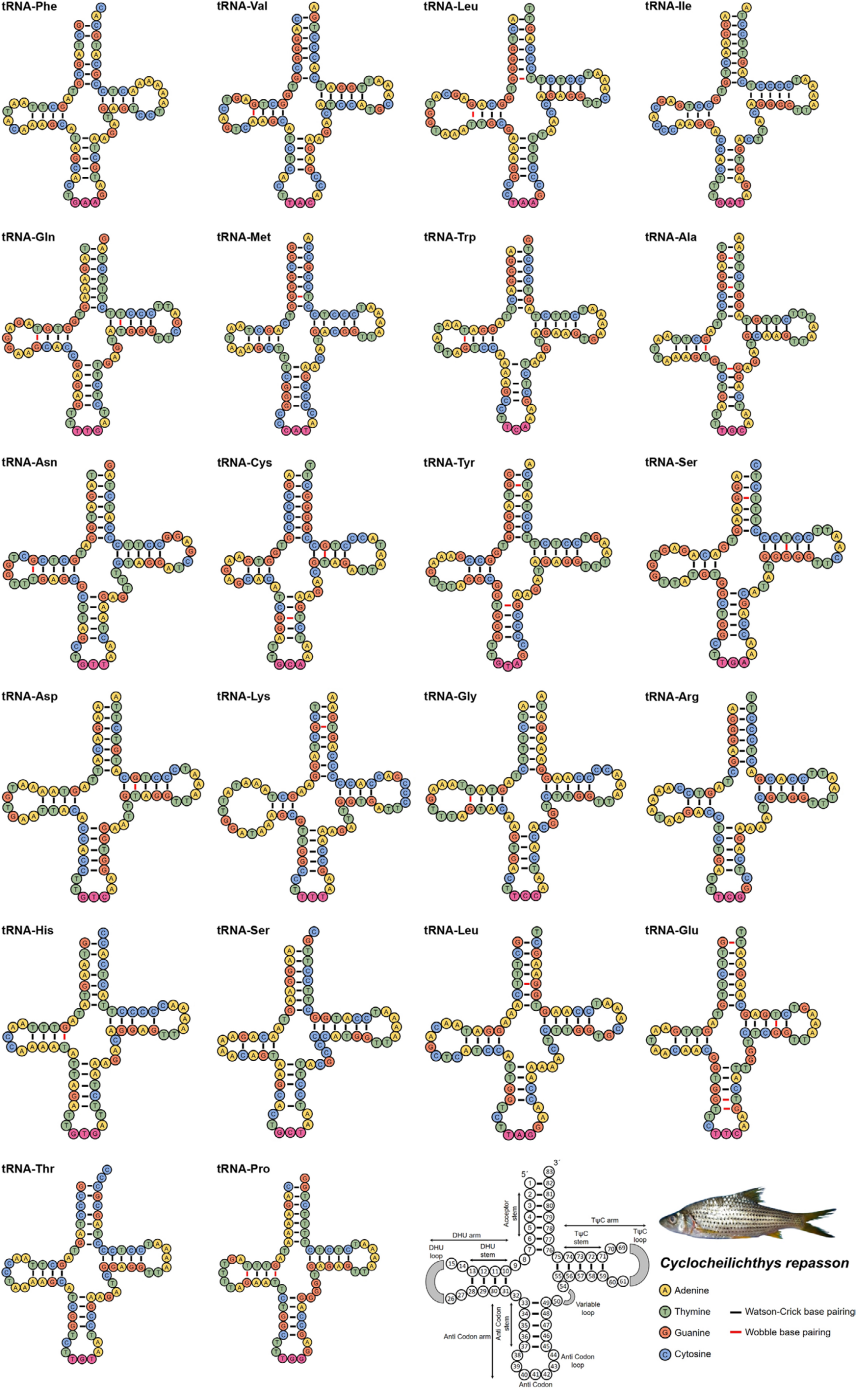
The secondary structures of the 22 transfer RNAs (tRNAs) within the *C. repasson* mitogenome display nucleotide composition and structural variability. Each tRNA is annotated with its three‐letter amino acid code, following the IUPAC‐IUB nomenclature. The final structure illustrates nucleotide positions and the stem‐loop configurations of the tRNAs. Watson‐Crick and wobble base pairings are indicated by black and red bars, respectively. The species photograph was taken by Mrs. Yossi Agusta (Government of Solok Regency, West Sumatra Province, Republic of Indonesia).

Focusing on rRNA genes, the *C. repasson* exhibited a total rRNA length of 2,587 bp, which is slightly shorter than that observed in *C. janthochir* (2636 bp), *C. apogon* (2634 bp), and *C. heteronema* (2634 bp). The nucleotide composition of the rRNA region in *C. repasson* was characterized by a relatively high A + T bias of 54.85%, accompanied by a distinct nucleotide bias reflected in an AT‐skew of 0.284 and a GC‐skew of −0.092. These values were generally consistent among congeners, with A + T bias ranging from 54.40% ( *C. apogon* ) to 55.77% ( *C. heteronema* ), AT‐skew values between 0.278 ( *C. heteronema* ) and 0.300 ( *C. janthochir* ), and GC‐skew ranging from −0.102 ( *C. janthochir* ) to −0.092 ( *C. repasson* ) (Table 2). Regarding tRNA genes, the *C. repasson* possessed a total length of 1,567 bp, slightly longer than *C. janthochir* (1563 bp) and *C. apogon* (1564 bp), but marginally shorter than *C. heteronema* (1568 bp). The A + T bias of the tRNA genes in *C. repasson* was 55.58%, with a positive AT‐skew of 0.121 and a negative GC‐skew of −0.126. This compositional pattern differs from that of its congeners, which presented lower AT‐skew values, ranging from 0.009 ( *C. heteronema* ) to 0.032 ( *C. apogon* ), and positive GC‐skew values between 0.055 ( *C. apogon* ) and 0.073 ( *C. heteronema* ) (Table 2). All 22 typical mitochondrial tRNA genes were successfully identified in *C. repasson* . The secondary structure predictions indicated that the majority adopted the standard cloverleaf conformation, characteristic of functional mitochondrial tRNAs. Each tRNA molecule exhibited the canonical structural elements, including the acceptor stem, dihydrouridine (DHU) arm, anticodon loop, and TΨC arm. Notably, the *tRNA‐Ser* (S1) displayed a simplified secondary structure due to the absence of base pairing in the DHU arm. Conversely, the *tRNA‐Leu* (L2) and *tRNA‐Lys* possessed elongated DHU arms with extended loop regions. The presence of both canonical Watson–Crick base pairings (A–T, G–C) and noncanonical wobble pairings (G–U) contributed to a structurally flexible yet functionally stable architecture. The G–U wobble base pairs were detected in multiple tRNAs of *C. repasson* , including *tRNA‐Val*, *tRNA‐Leu* (L2), *tRNA‐Gln*, *tRNA‐Trp*, *tRNA‐Ala*, *tRNA‐Asn*, *tRNA‐Cys*, *tRNA‐Tyr*, *tRNA‐Ser* (S2), *tRNA‐Asp*, *tRNA‐Lys*, *tRNA‐Gly*, *tRNA‐His*, *tRNA‐Leu* (L1), *tRNA‐Glu*, and *tRNA‐Pro*. These G–U pairings were distributed across various structural regions, including the acceptor stem, DHU arm, anticodon arm, and TΨC arm (Figure 4). Further analysis of the tRNA genes in *C. repasson* revealed that *tRNA‐Phe* featured the anticodon GAA, *tRNA‐Val* contained TAC, and *tRNA‐Leu* (L2) carried the anticodon TAA. The comparison of the tRNA anticodon sequences in *C. repasson* with those of the three congeners ( *C. janthochir* , *C. apogon* , and *C. heteronema* ) showed no variation, as all four species shared identical anticodon sequences across all 22 tRNA genes (Table S7).

### 3.6 Control Region Characteristics

**FIGURE 5:**
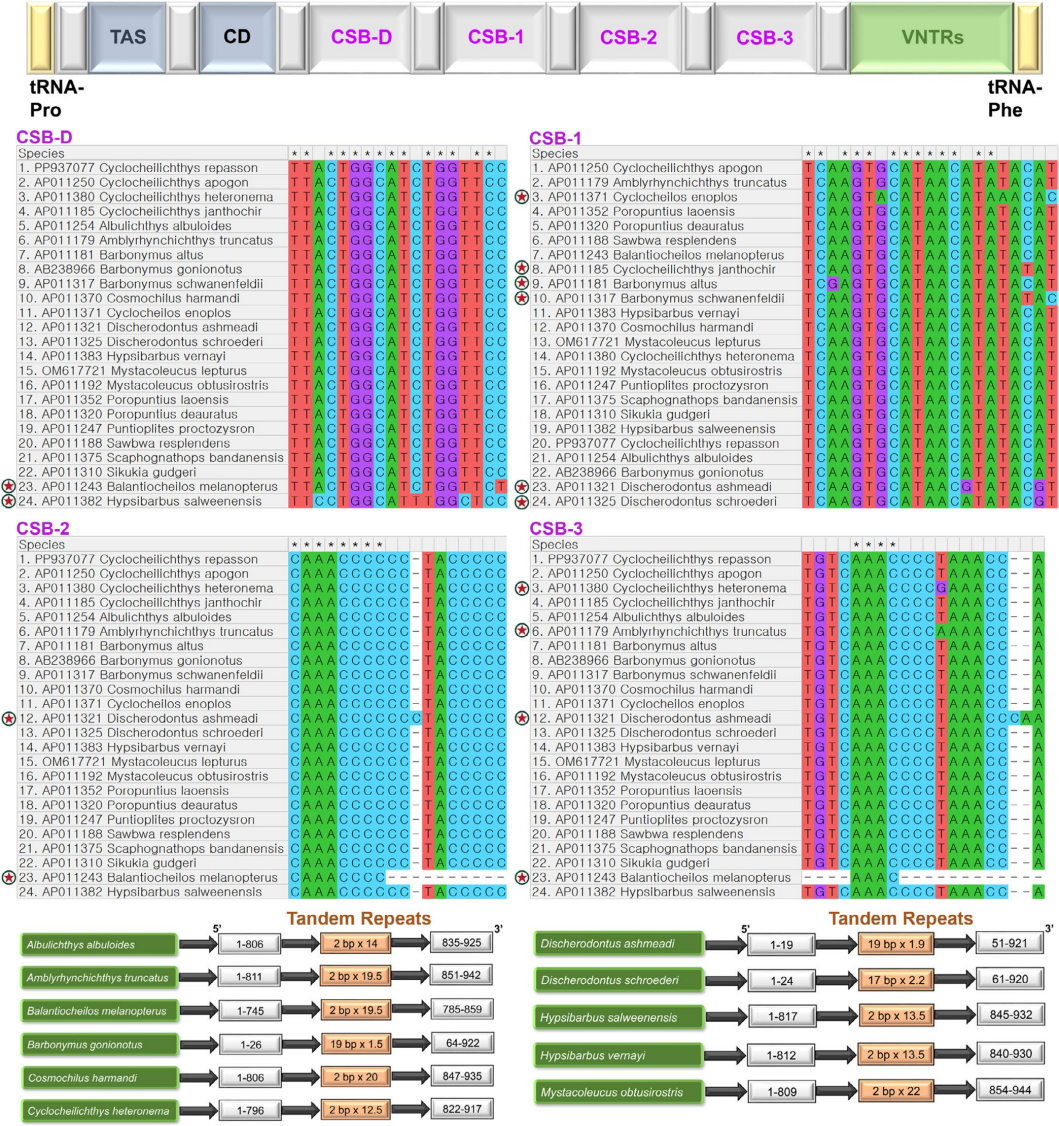
The schematic representation of conserved blocks within the CR of *C. repasson* and 23 other species within the “Poropuntiinae” clade. A conceptual linear overview of the CR is illustrated at the top. The conserved nucleotide positions across different “Poropuntiinae” species are indicated by black stars, whereas red stars denote species exhibiting nucleotide variations or deletions in sequence length. A detailed representation of the tandem repeats identified exclusively in 11 species within the “Poropuntiinae” clade is presented below.

The target species, *C. repasson* , was characterized by a CR length of 918 bp with a relatively high A + T bias of 68.74%. The nucleotide skewness analysis revealed a slightly positive AT‐skew value of 0.052 and a negative GC‐skew of −0.226. The comparative analysis with its congeners indicated that the CR length of *C. repasson* was intermediate, with *C. janthochir* and *C. heteronema* showing similar lengths of 926 and 917 bp, respectively, while *C. apogon* possessed the longest CR at 932 bp. The A + T bias in *C. repasson* was the highest among the species analyzed, followed by *C. heteronema* (68.59%), *C. janthochir* (67.39%), and lowest in *C. apogon* (66.09%). All species presented positive AT‐skew values, ranging from 0.011 in *C. heteronema* to 0.052 in both *C. repasson* and *C. apogon* . Conversely, all species showed negative GC‐skew values, with *C. repasson* and *C. janthochir* exhibiting values of −0.226 and −0.225, respectively, followed by *C. apogon* (−0.266), and the least skew observed in *C. heteronema* (−0.167) (Table 2). A detailed mitogenomic analysis of *C. repasson* alongside 23 other species within the “Poropuntiinae” clade identified four conserved sequence blocks (CSBs) within the mitochondrial CR, designated as CSB‐D, CSB‐1, CSB‐2, and CSB‐3. Although CSBs are typically regarded as highly conserved elements, the comparative analysis in this study demonstrated nucleotide length variations among these blocks, measuring 18 bp for CSB‐D and CSB‐2, 20 bp for CSB‐3, and 21 bp for CSB‐1. Several species within the clade exhibited unique nucleotide variations within these CSB regions. Specifically, the distinct nucleotide variants were detected in CSB‐D of *Balantiocheilos melanopterus* and *Hypsibarbus salweenensis* . The CSB‐1 region displayed nucleotide variations across six species: *C. enoplos*, *C. janthochir* , *Barbonymus altus* , *Barbonymus schwanenfeldii* , *Discherodontus ashmeadi* , and *Discherodontus schroederi* . The unique variations within CSB‐2 were observed in *D. ashmeadi* and *B. melanopterus* . Additionally, four species ( *C. heteronema* , *Amblyrhynchichthys truncatus* , *D. ashmeadi* , and *B. melanopterus* ) presented specific nucleotide variations within CSB‐3. Notably, *D. ashmeadi* displayed a higher cytosine content in CSB‐2 and a greater frequency of cytosine‐adenine dinucleotides in CSB‐3 compared to other species. Conversely, *B. melanopterus* possessed shorter sequence lengths in CSB‐2 and CSB‐3, characterized by the specific motifs “CAAACCCC” and “AAAC,” respectively. Furthermore, in CSB‐3, *C. heteronema* and *A. truncatus* showed decreased thymine content replaced by guanine and adenine, respectively, forming distinctive nucleotide patterns unique to these species. Significantly, among the 24 species analyzed, the tandem repeats within the CR were detected in only 11 species. The tandem repeats in eight species were predominantly composed of AT and TA base pairs. Meanwhile, *D. schroederi* presented a 17 bp tandem repeat with a frequency of 2.2 copies, *Barbonymus gonionotus* contained a 19 bp repeat repeated 1.5 times, and *D. ashmeadi* displayed a 19 bp tandem repeat occurring 1.9 times within the CR (Figure 5).

### 3.7 Phylogenetic Relationship of Clade “Poropuntiinae”

**FIGURE 6:**
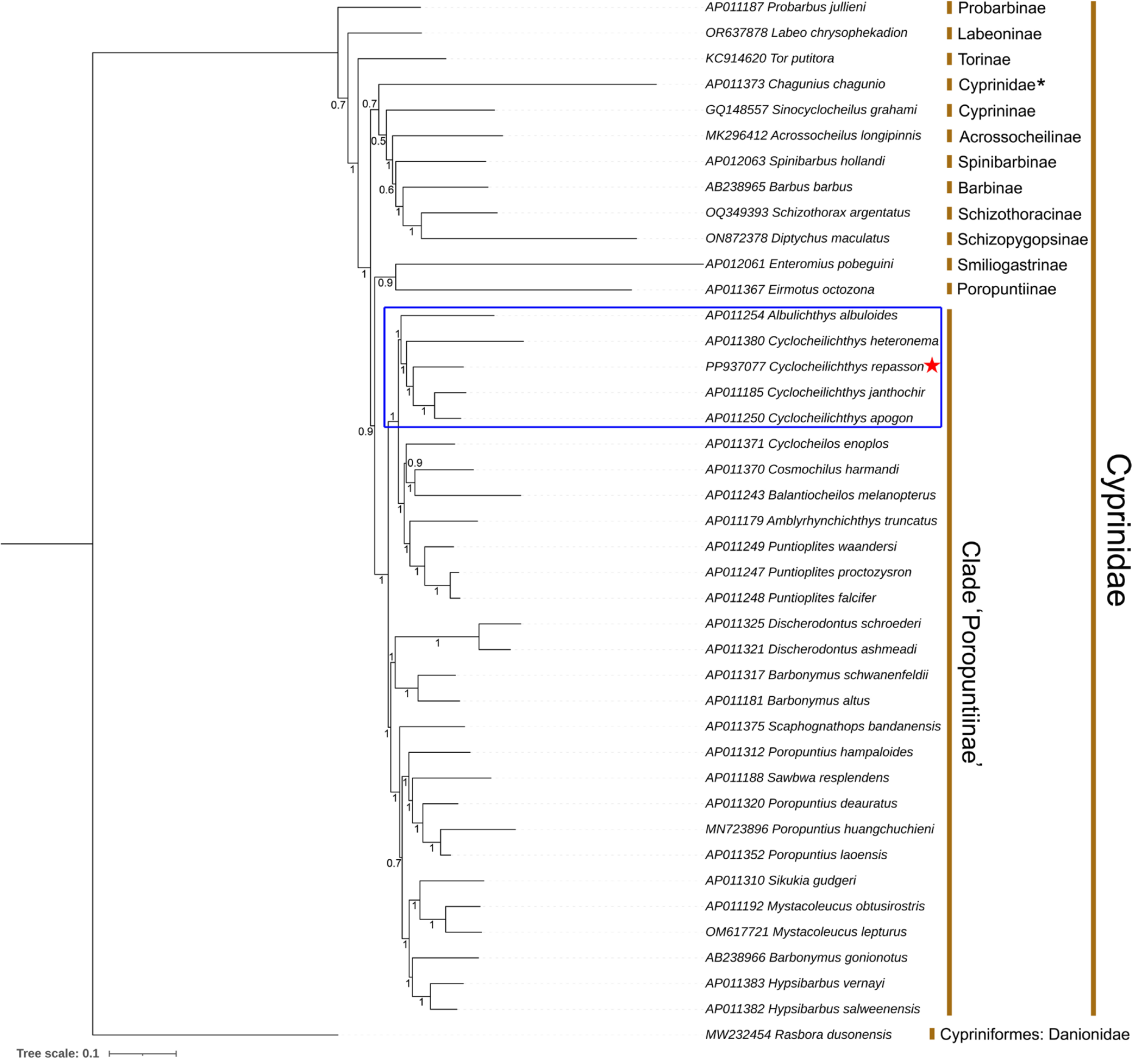
The Bayesian phylogenetic tree was constructed based on concatenated sequences of 13 PCGs, effectively distinguishing *C. repasson* from other species within the “Poropuntiinae” clade. The resulting cladogram offers insights into the evolutionary relationships across multiple taxonomic levels within the family Cyprinidae. The target species *C. repasson* is indicated by a red star, while the small black star represents species classified within the family Cyprinidae with an *incertae sedis* taxonomic status. The posterior probability support values are shown in red font at each corresponding node of the topology.

The phylogenetic analysis, conducted using both BA and ML approaches based on concatenated sequences of 13 PCGs, offers a comprehensive insight into the major phylogenetic relationships within the family Cyprinidae (Figure 6; Figure S1). The mitogenomic phylogenies revealed a close relationship between *Cyclocheilichthys* and *Albulichthys* in both BA and ML analyses. The congeners of *Cyclocheilichthys* formed a monophyletic clade, with *C. repasson* exhibiting a close phylogenetic affinity with *C. janthochir* and *C. apogon* . Overall, all species within the “Poropuntiinae” clade formed a well‐supported monophyletic group with high bootstrap values in the ML phylogeny (Figure S1). However, the BA inference showed a slightly different topology, where *E. octozona* clustered closely with *Enteromius pobeguini*, a member of the subfamily Smiliogastrinae. This unexpected association warrants further investigation into the phylogenetic positions of both species within their respective lineages (Figure 6). Interestingly, within the “Poropuntiinae” clade, *Sawbwa resplendens* further exhibited a contradictory placement, clustering with *Poropuntius* species. Additionally, the *Barbonymus* congeners were recovered as a paraphyletic group in both phylogenies, indicating a need for comprehensive systematic revision. Furthermore, the representative taxa from other cyprinid lineages, including *Labeo chrysophekadion* (Labeoninae), *Sinocyclocheilus grahami* (Cyprininae), *Acrossocheilus longipinnis* (Acrossocheilinae), *Barbus barbus* (Barbinae), *Probarbus jullieni* (Probarbinae), *Diptychus maculatus* (Schizopygopsinae), *Schizothorax argentatus* (Schizothoracinae), *Spinibarbus hollandi* (Spinibarbinae), *Tor putitora* (Torinae), as well as *C. chagunio* (Cyprinidae incertae sedis) were consistently placed as a distinct lineage in the present mitogenome‐based phylogenetic analyses (Figure 6; Figure S1).

### 3.8 Genetic Diversity and Phylogeny Based on *COI*

**FIGURE 7:**
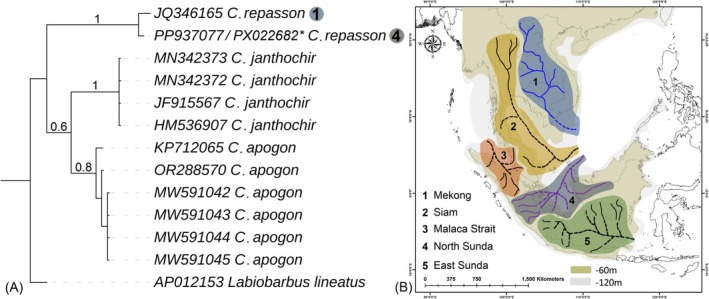
(A) The Bayesian tree constructed by partial *COI* gene sequences illustrates the phylogenetic relationships among *C. repasson* and its congeners. The posterior probability support values are indicated in black font at each corresponding node. The star symbol indicates the GenBank accession numbers (PX022682) of the partial *COI* sequence generated for *C. repasson* . The numbers “1” and “4” beside the clades indicate sequences originating from the Mekong and North Sunda river systems, respectively. (B) The close association of *C. repasson* populations from mainland and island regions of Southeast Asia corroborates the ancient Sundaland river system hypothesis. Specifically, the *C. repasson* from Lake Dibawah, Sumatra, Indonesia, an island ecosystem, exhibits affinity with the ancient North Sunda river system, whereas the population from Laos in mainland Southeast Asia is associated with the ancient Mekong river system. The different colors along with the numbers represent the ancient river systems in Southeast Asia.

The overall mean genetic distance within the *Cyclocheilichthys* dataset was 5.2% (ranging from 0% to 10.1%), including three congeners (Table S8). The mean intraspecific genetic distances for *C. repasson* and *C. apogon* were 0.7% and 0.3%, respectively. Notably, *C. repasson* exhibited mean interspecific genetic distances of 9.7% with *C. apogon* and 9.8% with *C. janthochir* . Additionally, a genetic distance of 4.5% was observed between *C. apogon* and *C. janthochir* . The *C. repasson* sequence generated from Sumatra, Indonesia, showed a genetic distance of 1.06% compared to sequences from mainland Laos. Both BA and ML phylogenies revealed cohesive clustering of the three *Cyclocheilichthys* species, each distinctly separated based on the partial *COI* gene (Figure 7A, Figure S2).

## 4 Discussion

### 4.1 Comparative Genetic Structure of *Cyclocheilichthys*

The mitogenome of *C. repasson* exhibits a conserved organization typical of teleost mitogenomes, consistent with previous findings across a broad range of freshwater fishes (Kundu, Kang, Go, et al. 2024; Kundu, Kang, Kim, et al. 2024). Several intergenic spacers were identified, with the longest (33 bp) located between *tRNA‐Asn* and *tRNA‐Cys*. In addition, overlapping regions were observed between *ND4L–ND4* and *ATP8–ATP6* (each spanning 7 bp), the latter of which is a well‐documented conserved feature in vertebrate mitogenomes, particularly among teleosts, where overlaps commonly range from 7 to 10 bp (Gomes‐dos‐Santos et al. 2023). The nucleotide composition and strand‐specific skews (AT‐skew and GC‐skew) varied among *Cyclocheilichthys* species, indicating lineage‐specific mutational pressures potentially driven by replication‐associated biases (Yoon et al. 2024). Notably, the *C. repasson* displayed an intermediate AT content compared to its congeners, with *C. janthochir* exhibiting the highest AT bias and *C. heteronema* the lowest. The elevated AT‐skew in *C. janthochir* may reflect transcriptional or replicative preferences for adenine‐rich motifs, whereas more balanced nucleotide distributions in *C. apogon* and *C. heteronema* may signal differences in mitochondrial genome stability or adaptive constraints (Aini et al. 2025). These compositional variations underscore the role of divergent mutational landscapes and evolutionary dynamics within the genus (Kundu, Kang, Go, et al. 2024; Kundu, Kang, Kim, et al. 2024). Overall, the bias toward A and T nucleotides in *Cyclocheilichthys* aligns with patterns commonly reported in teleost mitogenomes (Ewusi et al. 2024). The PCGs in *C. repasson* predominantly initiate with the standard ATG codon, except *COI*, which uses the alternative GTG start codon, a feature also present in other teleosts (Ojala et al. 1981). While initiation codons were largely conserved, the termination codons displayed greater variability. Six PCGs terminate with complete stop codons (TAA or TAG), whereas others employ incomplete stop codons (e.g., single thymine or dinucleotides), which are typically completed via posttranscriptional polyadenylation, a pattern conserved across *Cyclocheilichthys* species with minor interspecific differences (Jia and Higgs 2008). Significantly, the variations in termination codons in *COI*, *COIII*, and *ND4* among species may reflect subtle divergences in mitochondrial translational mechanisms (Kanaka et al. 2023).

The mitochondrial nucleotide diversity (π) varied considerably among PCGs, with some genes exhibiting higher polymorphism likely attributable to elevated mutation rates and potential adaptive evolution in teleosts (Duchêne et al. 2022). The saturation analysis revealed no evidence of substitution saturation, reaffirming the phylogenetic utility of these mitochondrial markers (Parvathy et al. 2022). The amino acid profiles across *Cyclocheilichthys* species were highly conserved, dominated by leucine, serine, threonine, and isoleucine—amino acids commonly favored due to codon usage bias and structural constraints in fish mitochondrial proteins (Yang and Nielsen 2000). The selective pressure analysis based on Ka/Ks ratios indicated strong purifying selection acting on all mitochondrial PCGs in *C. repasson* and its congeners, with all ratios below “1,” suggesting efficient elimination of deleterious mutations to preserve protein functionality (Nei and Kumar 2000; Wujdi et al. 2024). The ranking of selective constraint was as follows: *ND4L* < *COIII*<*COI* < *COII* < *ATP8* < *ATP6* < *ND3* < *ND4* < *ND1* < *ND6* < *ND5* < *ND2* < *Cytb*. The strong purifying selection observed in *ND4L* is likely attributed to its central role in the mitochondrial electron transport chain. The subunits of cytochrome oxidase (*COI–III*) and the ATP synthase (*ATP6*, *ATP8*) were also under strong evolutionary constraints (Hurst 2002). Conversely, the relatively relaxed selective pressure on *Cytb* and *ND2* implies faster evolutionary rates, in line with observations from other teleost mitochondrial genomes (Jacobsen et al. 2016).

The secondary structures of rRNA genes remained conserved across species, underscoring their essential roles in ribosomal assembly and mitochondrial translation (Bradshaw et al. 2005). All 22 tRNA genes displayed typical cloverleaf structures except *tRNA‐Ser* (S1), which lacked a stable DHU arm, a common deviation among metazoan mitogenomes. The lengths of tRNA genes varied, with *tRNA‐Leu* and *tRNA‐Lys* being the longest, and *tRNA‐Cys* the shortest—patterns consistent with other teleosts (Yoon et al. 2024). The presence of widespread G–U wobble base pairs suggests structural flexibility in the tRNA collection, likely enhancing codon recognition efficiency during translation (Salinas‐Giegé et al. 2015). The complete conservation of tRNA anticodon sequences across the studied *Cyclocheilichthys* species reflects their close evolutionary relationships and strong functional constraints on mitochondrial tRNA genes (Satoh et al. 2010; Fonseca et al. 2012). The species‐specific variations in CSBs, tandem repeat motifs, and key regulatory elements within the mitochondrial CR likely represent lineage‐specific modifications of its regulatory architecture (Ewusi et al. 2024; Aini et al. 2025). The tandem repeats in the CR showed variation in length, copy number, and positional distribution, suggesting differential replication slippage events and regulatory evolution across species (Lee et al. 1995). As hypervariable loci, the CR tandem repeats play critical roles not only in mitochondrial replication and transcription but also serve as robust molecular markers for high‐resolution analyses in fish species identification and population genetic studies (Lunt et al. 1998).

### 4.2 Evolutionary Relationships and Demographic Inferences

The phylogenetic reconstruction performed in this study reveals a close lineage association between *C. repasson* , *C. janthochir* , and *C. apogon* . Notably, these mitogenome‐based findings differ from previous phylogenies that utilized partial mitochondrial and nuclear markers (*Cytb*, *COI*, *RAG1*, and *RAG2*), which suggested a closer affinity between *C. repasson* and *C. armatus* (Pasco‐Viel et al. 2012; Yang et al. 2015). The absence of *C. armatus* in the current analysis, due to the unavailability of its mitogenomic data, likely underlies this divergence. Nevertheless, the mitogenome‐based phylogenetic approach employed here provides higher resolution and greater reliability compared to methods relying on limited gene fragments (Kundu, Kang, Go, et al. 2024; Kundu, Kang, Kim, et al. 2024). The morphological classifications also had previously grouped *C. repasson* and *C. apogon* as closely related taxa, while distinctly separating them from *C. enoplos* (Roberts 1989). Within *Cyclocheilichthys*, the presence of parallel sensory pore lines is a key diagnostic character absent in *C. enoplos* (Roberts 1989; Rainboth 1996; Kottelat 2001), further underscoring the taxonomic ambiguity of that species. Furthermore, several unexpected placements observed in the phylogenetic analyses highlight the need for further taxonomic and systematic investigation. Notably, *E. octozona* , traditionally placed within “Poropuntiinae,” clustered closely with *E. pobeguini*, a member of the subfamily Smiliogastrinae, in the BA phylogeny. This discordance suggests potential issues in current taxonomic assignments or the need for broader sampling and genetic data. Similarly, the *S. resplendens* exhibited an unusual phylogenetic position within the “Poropuntiinae” clade, grouping with *Poropuntius* species, contrary to its conventional placement. Additionally, *Barbonymus* congeners formed a paraphyletic group across the phylogenies, further indicating the necessity of a comprehensive systematic revision.

From a demographic perspective, the complex geology and paleogeographic history of the Sundaland biodiversity hotspot have been pivotal in shaping the distribution patterns and genetic structure of Southeast Asian freshwater fishes (Sholihah et al. 2021; Delrieu‐Trottin et al. 2025). The tectonic processes, including the subduction between the Asian and Australian plates, and intense volcanic activity, have profoundly altered habitats and established distinct biogeographic boundaries (Lohman et al. 2011). The physical barriers, such as deep‐sea trenches surrounding Sundaland, mountain ranges, and fault zones, have contributed to spatial isolation among populations, promoting genetic differentiation through limited dispersal, colonization bottlenecks, and habitat fragmentation (Šlechtová et al. 2025). The previous phylogeographic studies have presented cryptic lineages and high levels of genetic diversity in numerous freshwater species across this region, emphasizing the critical role of genetic data in biodiversity conservation planning (Sholihah et al. 2020; Wibowo et al. 2024). Notably, during the Last Glacial Maximum (approximately 26,500–19,000 years ago), sea levels dropped by about 120 m, exposing land bridges that facilitated ichthyofaunal exchanges between mainland and islands in Southeast Asia (Voris 2000; Cheng and Faidi 2025). However, this connectivity was disrupted during the Younger Dryas period (circa 12,500–11,500 years ago), when rising temperatures and a sea‐level increase of approximately 62 m gradually submerged lowlands, fragmenting Sundaland into its present‐day archipelagic configuration (Dansgaard et al. 1989; Lambeck et al. 2014; Harrington et al. 2024).

In this context, the limited availability of mitogenomic data has constrained comprehensive demographic reconstructions of *C. repasson* populations across mainland and island freshwater ecosystems in Southeast Asia. The partial *COI*‐based analysis revealed a 1.06% genetic divergence between *C. repasson* specimens sampled from Sumatra, Indonesia, and those from Laos, despite the geographic separation between mainland and island systems. These findings suggest the existence of possible cryptic diversity within *C. repasson* across Southeast Asia. It is plausible that populations of this species were historically connected through gene flow, potentially facilitated by paleo‐river systems during periods of lowered sea levels in the Sundaland region. Specifically, the *C. repasson* population may be linked to the paleo‐North Sunda river system, whereas the Laotian populations are likely connected to the paleo‐Mekong drainage (Figure 7B) (Voris 2000; Cheng and Faidi 2025). While this hypothesis enriches our understanding of *C. repasson* 's historical biogeography, extensive future studies with broader, more representative sampling across Southeast Asian habitats are essential. Such efforts are critical to elucidate the fine‐scale genetic diversity, population structure, and phylogeographic patterns necessary to validate informed conservation and management strategies for this freshwater fish species within the tropical biodiversity hotspot.

### 4.3 Conservation Framework of *C. repasson*

The target species, *C. repasson* , is currently classified as Least Concern by the IUCN due to its broad distribution and relatively stable populations. Nevertheless, the anthropogenic pressures such as habitat degradation and overfishing continue to pose substantial challenges to its long‐term viability (Vidthayanon and Lumbantobing 2020). As a freshwater species of both ecological and economic significance, *C. repasson* plays a vital role in maintaining ecosystem balance and sustaining the livelihoods of local communities in Southeast Asia. However, increasing levels of unregulated fishing may trigger localized population declines (Dudgeon et al. 2006). In Indonesia, *C. repasson* inhabits tectonic lakes in Sumatra, notably Lake Diatas (1462 m asl) and Lake Dibawah (1531 m asl), collectively referred to as the Twin Lakes (Siddiq et al. 2019). These freshwater ecosystems represent key components of biodiversity hotspots but are increasingly imperiled by intense anthropogenic activity. Such conditions necessitate the urgent implementation of evidence‐based conservation strategies to prevent further ecological degradation and conserve the native fish species (Lukman and Maghfiroh 2019). The advancements in molecular technologies, particularly genomics‐based approaches, provide powerful tools for accurate species identification, elucidation of phylogenetic relationships, and assessment of existing genetic diversity (Hinsinger et al. 2015). The present study utilizing the novel mitogenome of *C. repasson* and molecular evidence from the *COI* gene reveals preliminary indications of cryptic diversity and population differentiation between mainland (Laos) and island (Sumatra, Indonesia) ecosystems. Hence, these findings offer valuable insights into the genetic structure of this cyprinid species across Southeast Asia and underscore the importance of long‐term population monitoring and conservation planning (Mennesson et al. 2024).

### 4.4 Limitations and Future Directions

While this study presents the first complete mitogenome of the endemic freshwater fish *C. repasson* from Sundaland and elucidates its structural features and phylogenetic placement within the major Cyprinidae clade, there are notable limitations that should be addressed in future research. The present mitogenome‐based phylogenetic analysis included only four valid *Cyclocheilichthys* species ( *C. apogon* , *C. heteronema* , *C. janthochir* , and *C. repasson* ), while mitogenomic data for the remaining four valid species ( *C. armatus* , *C. lagleri* , *C. schoppeae*, and *C. sinensis* ) are currently unavailable. Thus, generating complete mitogenomes for these species is critical to provide a more comprehensive understanding of the matrilineal evolutionary relationships within this group. Furthermore, the genetic diversity estimation was based on only two *COI* sequences, one generated from a single specimen from Sumatra, Indonesia, and another retrieved from GenBank representing a specimen from Laos. This limited dataset may not adequately capture the full extent of genetic diversity or phylogeographic structure of *C. repasson* across its broader distribution in both mainland and island ecosystems of Southeast Asia. Therefore, future studies should involve extensive field sampling of this species across its geographic range, including multiple individuals from distinct populations, to better understand intraspecific variation. Overall, the enrichment of the molecular dataset by generating both mitochondrial and nuclear gene sequences will help clarify the population structure, gene flow, and demographic history of *C. repasson* in Southeast Asia.

## 5 Conclusion

This study successfully characterized the first complete mitogenome of *C. repasson* , providing valuable insights into its genomic architecture and phylogenetic placement. The genome length, nucleotide composition, codon usage, and gene organization exhibited highly conserved and consistent genomic features, aligning closely with those observed in other *Cyclocheilichthys* species. The phylogenetic analyses resolutely placed *C. repasson* within the “Poropuntiinae” clade, reinforcing current understanding of evolutionary relationships within the cyprinid group. In addition, the partial *COI* analysis revealed 1.06% genetic divergence in *C. repasson* between mainland (Laos) and island (Sumatra) populations, suggesting cryptic diversity likely shaped by paleo‐river systems during past sea‐level lows across the Sunda Shelf. However, broader and more representative sampling across diverse freshwater habitats in mainland and island Southeast Asia is needed to clarify fine‐scale genetic diversity, population structure, and phylogeographic patterns. Such an approach is critical for elucidating the genetic diversity and evolutionary dynamics of cyprinid species, thereby informing and enhancing conservation strategies within this tropical biodiversity hotspot.

## Author Contributions

**Ayu Fitri Izaki:** data curation (supporting), methodology (equal), writing – original draft (supporting). **Sarifah Aini:** formal analysis (equal), software (equal), writing – original draft (supporting). **Angkasa Putra:** formal analysis (equal), software (equal), writing – original draft (supporting). **Hey‐Eun Kang:** validation (equal), visualization (supporting). **Ah Ran Kim:** investigation (equal), methodology (equal). **Soo Rin Lee:** data curation (supporting), visualization (supporting). **Mugi Mulyono:** investigation (equal), validation (equal). **Muhammad Hilman Fu'adil Amin:** data curation (supporting), visualization (supporting). **Hyun‐Woo Kim:** conceptualization (equal), funding acquisition (equal), project administration (equal), resources (equal), supervision (equal), writing – review and editing (equal). **Shantanu Kundu:** conceptualization (equal), funding acquisition (equal), project administration (equal), resources (equal), supervision (equal), writing – review and editing (equal).

## Conflicts of Interest

The authors declare no conflicts of interest.

## Supporting information


**Figure S1:** The ML phylogenetic tree built using concatenated sequences of 13 PCGs, clearly delineates the newly sequenced *C. repasson* from other species within the “Poropuntiinae” clade. The resulting cladogram provides a detailed view of evolutionary relationships across various taxonomic ranks within the family Cyprinidae. The ML bootstrap values, shown in green circles at each node, represent the statistical confidence supporting each branch in the topology.
**Figure S2:** The ML phylogenetic tree inferred from *COI* gene sequences clearly delineates *C. repasson* from other *Cyclocheilichthys* congeners, demonstrating its distinct genetic divergence within the genus. The bootstrap support values, displayed in blue circles at each node, represent the statistical robustness of the corresponding branches in the topology. The star symbol indicates the GenBank accession numbers of the partial *COI* sequence generated for *C. repasson* .
**Table S1:** Mitogenome information for the newly sequenced *C. repasson* and other Cyprinidae species retrieved from GenBank for phylogenetic analyses.
**Table S2:** Mitochondrial *COI* sequence data for *C. repasson* obtained in this study and for other congeners retrieved from GenBank for phylogenetic analyses. The first serial number, with two accession numbers, refers to the sequence information of the complete mitogenome and the partial *COI* gene of *C. repasson* generated in the present study.
**Table S3:** Comparative analysis of intergenic nucleotide (IN) regions among the mitogenomes of four distinct *Cyclocheilichthys* species.
**Table S4:** *Comprehensive comparison of start and stop codons in PCGs across the mitogenomes of four Cyclocheilichthys species*.
**Table S5:** Comparative pairwise *K*_a_/*K*_s_ for each PCG across four *Cyclocheilichthys* species.
**Table S6:** The abundance of amino acids and RSCU values derived from the complete PCGs of four *Cyclocheilichthys* species.
**Table S7:** Detailed comparison of anticodon sequences present in tRNA genes across the mitogenomes of four *Cyclocheilichthys* species.
**Table S8:** Pairwise genetic distances among three *Cyclocheilichthys* species based on K2P model estimated from partial *COI* gene sequences.

## Data Availability

The mitogenome and partial *COI* sequence data supporting the findings of this study are publicly available in GenBank (NCBI) under the accession numbers PP937077 and PX022682, respectively, accessible at https://www.ncbi.nlm.nih.gov/.
