# Supplementary material for: Mitogenomic Profiling of Cyclocheilichthys repasson (Cypriniformes: Cyprinidae) and Its Phylogenetic Placement Within the Clade “Poropuntiinae”
Source: Ecol Evol. 2025 Aug 13;15(8):e71990. doi: 10.1002/ece3.71990 (PMC12350049; doi:10.1002/ece3.71990)
Supplement: Supplementary file 1 — Figure S1: The ML phylogenetic tree built using concatenated sequences of 13 PCGs, clearly delineates the newly sequenced C. repasson from other species within the “Poropuntiinae” clade. The resulting cladogram provides a detailed view of evolutionary relationships across various taxonomic ranks within the family Cyprinidae. The ML bootstrap values, shown in green circles at each node, represent the statistical confidence supporting each branch in the topology. Figure S2: The ML phylogenetic tree inferred from COI gene sequences clearly delineates C. repasson from other Cyclocheilichthys congeners, demonstrating its distinct genetic divergence within the genus. The bootstrap support values, displayed in blue circles at each node, represent the statistical robustness of the corresponding branches in the topology. The star symbol indicates the GenBank accession numbers of the partial COI sequence generated for C. repasson . Table S1: Mitogenome information for the newly sequenced C. repasson and other Cyprinidae species retrieved from GenBank for phylogenetic analyses. Table S2: Mitochondrial COI sequence data for C. repasson obtained in this study and for other congeners retrieved from GenBank for phylogenetic analyses. The first serial number, with two accession numbers, refers to the sequence information of the complete mitogenome and the partial COI gene of C. repasson generated in the present study. Table S3: Comparative analysis of intergenic nucleotide (IN) regions among the mitogenomes of four distinct Cyclocheilichthys species. Table S4: Comprehensive comparison of start and stop codons in PCGs across the mitogenomes of four Cyclocheilichthys species. Table S5: Comparative pairwise K a/K s for each PCG across four Cyclocheilichthys species. Table S6: The abundance of amino acids and RSCU values derived from the complete PCGs of four Cyclocheilichthys species. Table S7: Detailed comparison of anticodon sequences present in tRNA genes across the mitogenomes of [file ECE3-15-e71990-s001.docx]

**SUPPORTING INFORMATION**


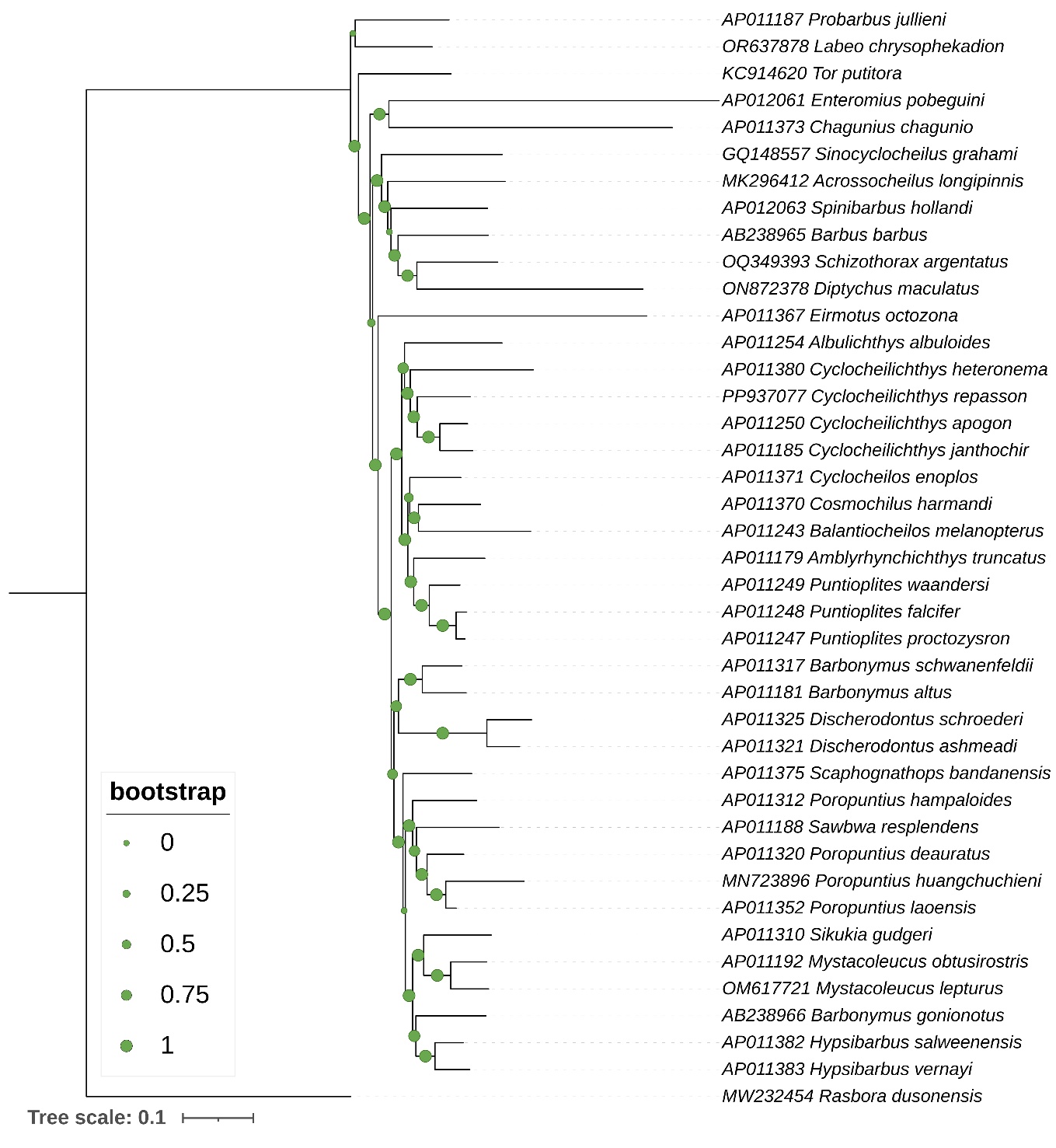


**Figure S1** The ML phylogenetic tree built using concatenated sequences of 13 PCGs, clearly delineating the newly sequenced *C. repasson* from other species within the ‘Poropuntiinae’ clade. The resulting cladogram provides a detailed view of evolutionary relationships across various taxonomic ranks within the family Cyprinidae. The ML bootstrap values, shown in green circle at each node, represent the statistical confidence supporting each branch in the topology.


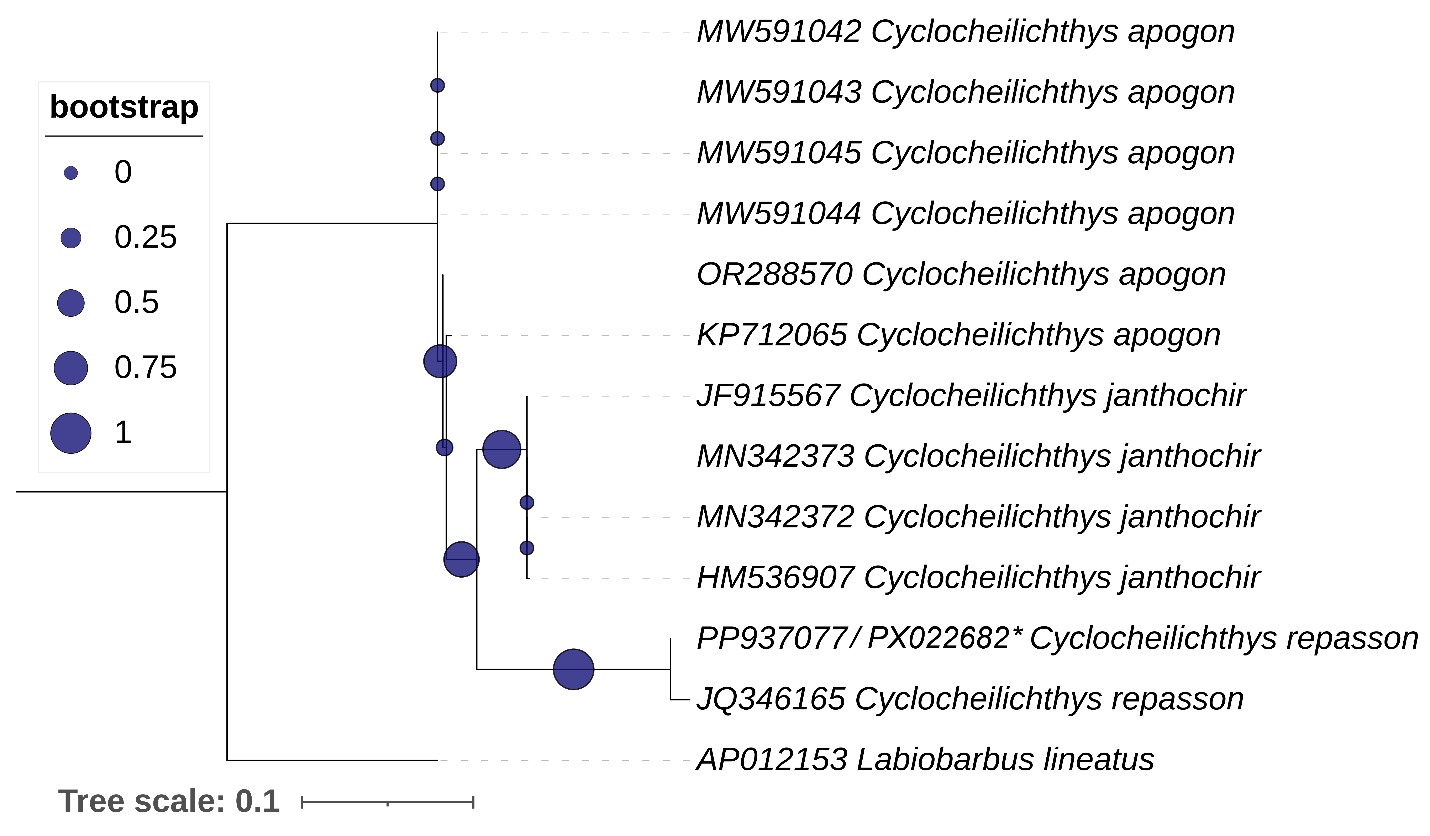


**Figure S2** The ML phylogenetic tree inferred from *COI* gene sequences clearly delineates *C. repasson* from other *Cyclocheilichthys* congeners, demonstrating its distinct genetic divergence within the genus. The bootstrap support values, displayed in blue circle at each node, represent the statistical robustness of the corresponding branches in the topology. The star symbol indicates the GenBank accession numbers of the partial *COI* sequence generated for *C. repasson*.

**Table S1** Mitogenome information for the newly sequenced *C. repasson* and other Cyprinidae species retrieved from GenBank for phylogenetic analyses.

| **Sl. No.** | **Taxonomic Rank** | **GenBank Name** | **Valid Species Name** | **Accession No.** |
| --- | --- | --- | --- | --- |
| 1 | ‘Poropuntiinae’ | *Cyclocheilichthys repasson* | *Cyclocheilichthys repasson* | PP937077 |
| 2 | ‘Poropuntiinae’ | *Albulichthys albuloides* | *Albulichthys albuloides* | AP011254 |
| 3 | ‘Poropuntiinae’ | *Amblyrhynchichthys truncatus* | *Amblyrhynchichthys truncatus* | AP011179 |
| 4 | ‘Poropuntiinae’ | *Cyclocheilichthys apogon* | *Cyclocheilichthys apogon* | AP011250 |
| 5 | ‘Poropuntiinae’ | *Balantiocheilos melanopterus* | *Balantiocheilos melanopterus* | AP011243 |
| 6 | ‘Poropuntiinae’ | *Barbonymus altus* | *Barbonymus altus* | AP011181 |
| 7 | ‘Poropuntiinae’ | *Barbonymus gonionotus* | *Barbonymus gonionotus* | AB238966 |
| 8 | ‘Poropuntiinae’ | *Barbonymus schwanenfeldii* | *Barbonymus schwanenfeldii* | AP011317 |
| 9 | ‘Poropuntiinae’ | *Cosmochilus harmandi* | *Cosmochilus harmandi* | AP011370 |
| 10 | ‘Poropuntiinae’ | *Cyclocheilichthys enoplos* | *Cyclocheilos enoplos* | AP011371 |
| 11 | ‘Poropuntiinae’ | *Cyclocheilichthys heteronema* | *Cyclocheilichthys heteronema* | AP011380 |
| 12 | ‘Poropuntiinae’ | *Cyclocheilichthys janthochir* | *Cyclocheilichthys janthochir* | AP011185 |
| 13 | ‘Poropuntiinae’ | *Discherodontus ashmeadi* | *Discherodontus ashmeadi* | AP011321 |
| 14 | ‘Poropuntiinae’ | *Discherodontus schroederi* | *Discherodontus schroederi* | AP011325 |
| 15 | ‘Poropuntiinae’ | *Eirmotus octozona* | *Eirmotus octozona* | AP011367 |
| 16 | ‘Poropuntiinae’ | *Hypsibarbus salweenensis* | *Hypsibarbus salweenensis* | AP011382 |
| 17 | ‘Poropuntiinae’ | *Hypsibarbus vernayi* | *Hypsibarbus vernayi* | AP011383 |
| 18 | ‘Poropuntiinae’ | *Mystacoleucus lepturus* | *Mystacoleucus lepturus* | OM617721 |
| 19 | ‘Poropuntiinae’ | *Mystacoleucus marginatus* | *Mystacoleucus obtusirostris* | AP011192 |
| 20 | ‘Poropuntiinae’ | *Poropuntius bantamensis* | *Poropuntius laoensis* | AP011352 |
| 21 | ‘Poropuntiinae’ | *Poropuntius huangchuchieni* | *Poropuntius huangchuchieni* | MN723896 |
| 22 | ‘Poropuntiinae’ | *Poropuntius normani* | *Poropuntius deauratus* | AP011320 |
| 23 | ‘Poropuntiinae’ | *Poropuntius scapanognathus* | *Poropuntius hampaloides* | AP011312 |
| 24 | ‘Poropuntiinae’ | *Puntioplites falcifer* | *Puntioplites falcifer* | AP011248 |
| 25 | ‘Poropuntiinae’ | *Puntioplites proctozysron* | *Puntioplites proctozysron* | AP011247 |
| 26 | ‘Poropuntiinae’ | *Puntioplites waandersi* | *Puntioplites waandersi* | AP011249 |
| 27 | ‘Poropuntiinae’ | *Sawbwa resplendens* | *Sawbwa resplendens* | AP011188 |
| 28 | ‘Poropuntiinae’ | *Scaphognathops bandanensis* | *Scaphognathops bandanensis* | AP011375 |
| 29 | ‘Poropuntiinae’ | *Sikukia gudgeri* | *Sikukia gudgeri* | AP011310 |
| 30 | Labeoninae | *Labeo chrysophekadion* | *Labeo chrysophekadion* | OR637878 |
| 31 | Cyprininae | *Sinocyclocheilus grahami* | *Sinocyclocheilus grahami* | GQ148557 |
| 32 | Acrossocheilinae | *Acrossocheilus longipinnis* | *Acrossocheilus longipinnis* | MK296412 |
| 33 | Barbinae | *Barbus barbus* | *Barbus barbus* | AB238965 |
| 34 | Probarbinae | *Probarbus jullieni* | *Probarbus jullieni* | AP011187 |
| 35 | Schizopygopsinae | *Diptychus maculatus* | *Diptychus maculatus* | ON872378 |
| 36 | Schizothoracinae | *Schizothorax argentatus* | *Schizothorax argentatus* | OQ349393 |
| 37 | Smiliogastrinae | *Enteromius pobeguini* | *Enteromius pobeguini* | AP012061 |
| 38 | Spinibarbinae | *Spinibarbus hollandi* | *Spinibarbus hollandi* | AP012063 |
| 39 | Torinae | *Tor putitora* | *Tor putitora* | KC914620 |
| 40 | Cyprinidae incertae sedis | *Chagunius chagunio* | *Chagunius chagunio* | AP011373 |
| 41 | Cypriniformes: Danionidae | *Rasbora dusonensis* | *Rasbora dusonensis* | MW232454 |

**Table S2** Mitochondrial *COI* sequence data for *C. repasson* obtained in this study and for other congeners retrieved from GenBank for phylogenetic analyses. The first serial number, with two accession numbers, refers to the sequence information of the complete mitogenome and the partial *COI* gene of *C. repasson* generated in the present study.

| **Sl. No.** | **Accession No.** | **GenBank Name** | **Valid Species Name** | **Locality** |
| --- | --- | --- | --- | --- |
| 1 | PP937077/ PX022682 | Cyclocheilichthys repasson | *Cyclocheilichthys repasson* | Sumatra, Indonesia |
| 2 | JQ346165 | *Anematichthys repasson* | *Cyclocheilichthys repasson* | Laos |
| 3 | MN342373 | *Cyclocheilichthys janthochir* | *Cyclocheilichthys janthochir* | Aquarium trade |
| 4 | MN342372 | *Cyclocheilichthys janthochir* | *Cyclocheilichthys janthochir* | Aquarium trade |
| 5 | JF915567 | *Cyclocheilichthys janthochir* | *Cyclocheilichthys janthochir* | Aquarium trade |
| 6 | HM536907 | *Cyclocheilichthys janthochir* | *Cyclocheilichthys janthochir* | - |
| 7 | OR288570 | *Cyclocheilichthys apogon* | *Cyclocheilichthys apogon* | Indonesia |
| 8 | MW591045 | *Cyclocheilichthys apogon* | *Cyclocheilichthys apogon* | Malaysia |
| 9 | MW591044 | *Cyclocheilichthys apogon* | *Cyclocheilichthys apogon* | Malaysia |
| 10 | MW591043 | *Cyclocheilichthys apogon* | *Cyclocheilichthys apogon* | Malaysia |
| 11 | MW591042 | *Cyclocheilichthys apogon* | *Cyclocheilichthys apogon* | Malaysia |
| 12 | KP712065 | *Anematichthys apogon* | *Cyclocheilichthys apogon* | Cambodia |
| 13 | AP012153 | *Labiobarbus lineatus* | *Labiobarbus lineatus* | Outgroup |

**Table S3** Comparative analysis of intergenic nucleotide (IN) regions among the mitogenomes of four distinct *Cyclocheilichthys* species.

| **Genes** | ***C. repasson*** | | | ***C. jantochir*** | | | ***C. apogon*** | | | ***C. heteronema*** | | |
| --- | --- | --- | --- | --- | --- | --- | --- | --- | --- | --- | --- | --- |
|  | **Start** | **End** | **IN** | **Start** | **End** | **IN** | **Start** | **End** | **IN** | **Start** | **End** | **IN** |
| *tRNA-Phe* | 1 | 69 | 0 | 1 | 69 | 0 | 1 | 69 | 0 | 1 | 69 | 0 |
| *12S rRNA* | 70 | 1022 | 0 | 70 | 1026 | 0 | 70 | 1023 | 0 | 70 | 1022 | 0 |
| *tRNA-Val* | 1023 | 1094 | 0 | 1027 | 1098 | 0 | 1024 | 1095 | 0 | 1023 | 1094 | 0 |
| *16S rRNA* | 1095 | 2775 | 0 | 1099 | 2777 | 0 | 1096 | 2775 | 0 | 1095 | 2775 | 0 |
| *tRNA-Leu* | 2776 | 2851 | 0 | 2778 | 2853 | 0 | 2776 | 2851 | 0 | 2776 | 2851 | 0 |
| *ND1* | 2852 | 3826 | 4 | 2854 | 3828 | 5 | 2852 | 3826 | 5 | 2852 | 3826 | 4 |
| *tRNA-Ile* | 3831 | 3902 | -2 | 3834 | 3905 | -2 | 3832 | 3903 | -2 | 3831 | 3902 | -2 |
| *tRNA-Gln* | 3901 | 3971 | 1 | 3904 | 3974 | 1 | 3902 | 3972 | 1 | 3901 | 3971 | 1 |
| *tRNA-Met* | 3973 | 4041 | 0 | 3976 | 4044 | 0 | 3974 | 4042 | 0 | 3973 | 4041 | 0 |
| *ND2* | 4042 | 5086 | 0 | 4045 | 5089 | 0 | 4043 | 5087 | 0 | 4042 | 5086 | 0 |
| *tRNA-Trp* | 5087 | 5157 | 2 | 5090 | 5160 | 2 | 5088 | 5158 | 2 | 5087 | 5159 | 2 |
| *tRNA-Ala* | 5160 | 5228 | 1 | 5163 | 5231 | 2 | 5161 | 5229 | 1 | 5162 | 5230 | 1 |
| *tRNA-Asn* | 5230 | 5302 | 33 | 5234 | 5305 | 33 | 5231 | 5303 | 33 | 5232 | 5304 | 32 |
| *tRNA-Cys* | 5336 | 5402 | -1 | 5339 | 5405 | -1 | 5337 | 5403 | -1 | 5337 | 5403 | -1 |
| *tRNA-Tyr* | 5402 | 5472 | 1 | 5405 | 5475 | 1 | 5403 | 5473 | 1 | 5403 | 5472 | 1 |
| *COI* | 5474 | 7021 | -1 | 5477 | 7027 | 0 | 5475 | 7025 | 0 | 5474 | 7030 | -8 |
| *tRNA-Ser* | 7021 | 7092 | 3 | 7028 | 7098 | 1 | 7026 | 7096 | 3 | 7023 | 7093 | 2 |
| *tRNA-Asp* | 7096 | 7167 | 14 | 7100 | 7171 | 13 | 7100 | 7171 | 13 | 7096 | 7167 | 16 |
| *COII* | 7182 | 7872 | 0 | 7185 | 7875 | 0 | 7185 | 7875 | 0 | 7184 | 7874 | 0 |
| *tRNA-Lys* | 7873 | 7948 | 1 | 7876 | 7951 | 1 | 7876 | 7951 | 1 | 7875 | 7950 | 2 |
| *ATP8* | 7950 | 8114 | -7 | 7953 | 8117 | -7 | 7953 | 8117 | -7 | 7953 | 8117 | -7 |
| *ATP6* | 8108 | 8790 | 0 | 8111 | 8793 | 0 | 8111 | 8793 | 0 | 8111 | 8793 | 0 |
| *COIII* | 8791 | 9575 | 0 | 8794 | 9578 | -1 | 8794 | 9577 | 0 | 8794 | 9578 | 0 |
| *tRNA-Gly* | 9576 | 9648 | 0 | 9578 | 9650 | 0 | 9578 | 9650 | 0 | 9579 | 9651 | 0 |
| *ND3* | 9649 | 9997 | 0 | 9651 | 9999 | 0 | 9651 | 9999 | 0 | 9652 | 10000 | 0 |
| *tRNA-Arg* | 9998 | 10067 | 0 | 10000 | 10069 | 0 | 10000 | 10069 | 0 | 10001 | 10072 | 0 |
| *ND4L* | 10068 | 10364 | -7 | 10070 | 10366 | -7 | 10070 | 10366 | -7 | 10073 | 10369 | -7 |
| *ND4* | 10358 | 11738 | 0 | 10360 | 11740 | 0 | 10360 | 11740 | 0 | 10363 | 11743 | 0 |
| *tRNA-His* | 11739 | 11807 | 0 | 11741 | 11809 | 0 | 11741 | 11809 | 0 | 11744 | 11812 | 0 |
| *tRNA-Ser* | 11808 | 11877 | 1 | 11810 | 11878 | 1 | 11810 | 11878 | 1 | 11813 | 11882 | 1 |
| *tRNA-Leu* | 11879 | 11951 | 3 | 11880 | 11952 | 4 | 11880 | 11952 | 4 | 11884 | 11956 | 2 |
| *ND5* | 11955 | 13778 | -4 | 11957 | 13780 | -4 | 11957 | 13780 | -4 | 11959 | 13782 | -4 |
| *ND6* | 13775 | 14296 | 0 | 13777 | 14298 | 0 | 13777 | 14298 | 0 | 13779 | 14300 | 0 |
| *tRNA-Glu* | 14297 | 14365 | 5 | 14299 | 14367 | 5 | 14299 | 14367 | 5 | 14301 | 14369 | 5 |
| *Cytb* | 14371 | 15511 | 0 | 14373 | 15513 | 0 | 14373 | 15513 | 0 | 14375 | 15515 | 0 |
| *tRNA-Thr* | 15512 | 15583 | -1 | 15514 | 15585 | -1 | 15514 | 15585 | -1 | 15516 | 15587 | -1 |
| *tRNA-Pro* | 15583 | 15653 | 0 | 15585 | 15654 | 0 | 15585 | 15654 | 0 | 15587 | 15656 | 0 |
| *Control region* | 15654 | 16571 | - | 15655 | 16580 | - | 15655 | 16586 | - | 15657 | 16573 | - |

**Table S4** Comprehensive comparison of start and stop codons in PCGs across the mitogenomes of four *Cyclocheilichthys* species.

| **Genes** | ***C. repasson*** | | ***C. jantochir*** | | ***C. apogon*** | | ***C. heteronema*** | |
| --- | --- | --- | --- | --- | --- | --- | --- | --- |
|  | **Start** | **Stop** | **Start** | **Stop** | **Start** | **Stop** | **Start** | **Stop** |
| *ND1* | ATG | TAA | ATG | TAA | ATG | TAA | ATG | TAA |
| *ND2* | ATG | T-- | ATG | TAG | ATG | TAG | ATG | TAA |
| *COI* | GTG | TAA | GTG | TAA | GTG | TAA | GTG | AGG |
| *COII* | ATG | T-- | ATG | T-- | ATG | T-- | ATG | T-- |
| *ATP8* | ATG | TAG | ATG | TAG | ATG | TAG | ATG | TAG |
| *ATP6* | ATG | TA- | ATG | TA- | ATG | TTA | ATG | TTA |
| *COIII* | ATG | TA- | ATG | TA- | ATG | T-- | ATG | TA- |
| *ND3* | ATG | T-- | ATG | T-- | ATG | T-- | ATG | T-- |
| *ND4L* | ATG | TAA | ATG | TAA | ATG | TAA | ATG | TAA |
| *ND4* | ATG | T-- | ATG | T-- | ATG | T-- | ATG | TT- |
| *ND5* | ATG | TAA | ATG | TAA | ATG | TAA | ATG | TAA |
| *ND6* | ATG | TAA | ATG | TAA | ATG | TAA | ATG | TAA |
| *Cytb* | ATG | TT- | ATG | TT- | ATG | TT- | ATG | T-- |

**Table S5** Comparative pairwise Ka/Ks for each PCG across four *Cyclocheilichthys* species.

| **Genes** | ***NAD1*** | ***NAD2*** | ***COI*** | ***COII*** | ***ATP8*** | ***ATP6*** | ***COIII*** | ***NAD3*** | ***NAD4L*** | ***NAD4*** | ***NAD5*** | ***NAD6*** | ***Cytb*** |
| --- | --- | --- | --- | --- | --- | --- | --- | --- | --- | --- | --- | --- | --- |
|  | 0.040 | 0.053 | 0.008 | 0.014 | 0.000 | 0.017 | 0.005 | 0.018 | 0.010 | 0.058 | 0.062 | 0.041 | 0.063 |
|  | 0.053 | 0.040 | 0.010 | 0.009 | 0.000 | 0.028 | 0.010 | 0.040 | 0.010 | 0.024 | 0.047 | 0.030 | 0.048 |
|  | 0.040 | 0.097 | 0.017 | 0.039 | 0.066 | 0.024 | 0.017 | 0.050 | 0.008 | 0.032 | 0.068 | 0.068 | 0.095 |
| **Average** | 0.0443 | 0.0633 | 0.0117 | 0.0207 | 0.0220 | 0.0230 | 0.0107 | 0.0360 | 0.0093 | 0.0380 | 0.0590 | 0.0463 | 0.0687 |
| **STDEV** | 0.0075 | 0.0299 | 0.0047 | 0.0161 | 0.0381 | 0.0056 | 0.0060 | 0.0164 | 0.0012 | 0.0178 | 0.0108 | 0.0196 | 0.0240 |

**Table S6** The abundance of amino acids and RSCU values derived from the complete PCGs of four *Cyclocheilichthys* species.

| ***C. repasson*** | | | | | | | | | | | |
| --- | --- | --- | --- | --- | --- | --- | --- | --- | --- | --- | --- |
| Codon | Count | RSCU | Codon | Count | RSCU | Codon | Count | RSCU | Codon | Count | RSCU |
| UUU(F) | 59 | 0.98 | UCU(S) | 63 | 0.98 | UAU(Y) | 105 | 1.12 | UGU(C) | 35 | 0.96 |
| UUC(F) | 62 | 1.02 | UCC(S) | 82 | 1.28 | UAC(Y) | 82 | 0.88 | UGC(C) | 38 | 1.04 |
| UUA(L) | 101 | 1.36 | UCA(S) | 75 | 1.17 | UAA(*) | 110 | 1.41 | UGA(*) | 50 | 0.64 |
| UUG(L) | 46 | 0.62 | UCG(S) | 33 | 0.52 | UAG(*) | 74 | 0.95 | UGG(W) | 45 | 1.00 |
| CUU(L) | 79 | 1.06 | CCU(P) | 102 | 1.21 | CAU(H) | 83 | 1.01 | CGU(R) | 29 | 0.78 |
| CUC(L) | 51 | 0.69 | CCC(P) | 90 | 1.07 | CAC(H) | 82 | 0.99 | CGC(R) | 29 | 0.78 |
| CUA(L) | 122 | 1.64 | CCA(P) | 115 | 1.36 | CAA(Q) | 96 | 1.21 | CGA(R) | 29 | 0.78 |
| CUG(L) | 47 | 0.63 | CCG(P) | 30 | 0.36 | CAG(Q) | 63 | 0.79 | CGG(R) | 26 | 0.70 |
| AUU(I) | 111 | 1.29 | ACU(T) | 115 | 1.28 | AAU(N) | 113 | 1.05 | AGU(S) | 50 | 0.78 |
| AUC(I) | 76 | 0.88 | ACC(T) | 100 | 1.11 | AAC(N) | 102 | 0.95 | AGC(S) | 81 | 1.27 |
| AUA(I) | 71 | 0.83 | ACA(T) | 107 | 1.19 | AAA(K) | 76 | 1.49 | AGA(R) | 52 | 1.40 |
| AUG(M) | 51 | 1.00 | ACG(T) | 38 | 0.42 | AAG(K) | 26 | 0.51 | AGG(R) | 58 | 1.56 |
| GUU(V) | 15 | 0.85 | GCU(A) | 22 | 0.68 | GAU(D) | 37 | 1.01 | GGU(G) | 11 | 0.49 |
| GUC(V) | 10 | 0.56 | GCC(A) | 55 | 1.71 | GAC(D) | 36 | 0.99 | GGC(G) | 22 | 0.98 |
| GUA(V) | 28 | 1.58 | GCA(A) | 48 | 1.49 | GAA(E) | 46 | 1.19 | GGA(G) | 34 | 1.51 |
| GUG(V) | 18 | 1.01 | GCG(A) | 4 | 0.12 | GAG(E) | 31 | 0.81 | GGG(G) | 23 | 1.02 |
| ***C. janthochir*** | | | | | | | | | | | |
| Codon | Count | RSCU | Codon | Count | RSCU | Codon | Count | RSCU | Codon | Count | RSCU |
| UUU(F) | 58 | 1.06 | UCU(S) | 70 | 1.07 | UAU(Y) | 117 | 1.08 | UGU(C) | 31 | 1.13 |
| UUC(F) | 51 | 0.94 | UCC(S) | 88 | 1.34 | UAC(Y) | 99 | 0.92 | UGC(C) | 24 | 0.87 |
| UUA(L) | 94 | 1.27 | UCA(S) | 79 | 1.20 | UAA(*) | 110 | 1.37 | UGA(*) | 53 | 0.66 |
| UUG(L) | 49 | 0.66 | UCG(S) | 31 | 0.47 | UAG(*) | 78 | 0.97 | UGG(W) | 38 | 1.00 |
| CUU(L) | 73 | 0.99 | CCU(P) | 97 | 1.23 | CAU(H) | 83 | 1.03 | CGU(R) | 29 | 0.76 |
| CUC(L) | 54 | 0.73 | CCC(P) | 77 | 0.98 | CAC(H) | 78 | 0.97 | CGC(R) | 29 | 0.76 |
| CUA(L) | 129 | 1.75 | CCA(P) | 113 | 1.43 | CAA(Q) | 96 | 1.25 | CGA(R) | 23 | 0.60 |
| CUG(L) | 44 | 0.60 | CCG(P) | 28 | 0.36 | CAG(Q) | 57 | 0.75 | CGG(R) | 33 | 0.86 |
| AUU(I) | 114 | 1.23 | ACU(T) | 108 | 1.23 | AAU(N) | 116 | 1.01 | AGU(S) | 48 | 0.73 |
| AUC(I) | 87 | 0.94 | ACC(T) | 106 | 1.21 | AAC(N) | 113 | 0.99 | AGC(S) | 78 | 1.19 |
| AUA(I) | 78 | 0.84 | ACA(T) | 103 | 1.17 | AAA(K) | 84 | 1.53 | AGA(R) | 52 | 1.36 |
| AUG(M) | 57 | 1.00 | ACG(T) | 34 | 0.39 | AAG(K) | 26 | 0.47 | AGG(R) | 64 | 1.67 |
| GUU(V) | 17 | 0.99 | GCU(A) | 18 | 0.60 | GAU(D) | 27 | 0.75 | GGU(G) | 13 | 0.59 |
| GUC(V) | 8 | 0.46 | GCC(A) | 50 | 1.67 | GAC(D) | 45 | 1.25 | GGC(G) | 19 | 0.86 |
| GUA(V) | 31 | 1.80 | GCA(A) | 48 | 1.60 | GAA(E) | 42 | 1.17 | GGA(G) | 38 | 1.73 |
| GUG(V) | 13 | 0.75 | GCG(A) | 4 | 0.13 | GAG(E) | 30 | 0.83 | GGG(G) | 18 | 0.82 |
| ***C. apogon*** | | | | | | | | | | | |
| Codon | Count | RSCU | Codon | Count | RSCU | Codon | Count | RSCU | Codon | Count | RSCU |
| UUU(F) | 60 | 1.09 | UCU(S) | 64 | 0.98 | UAU(Y) | 116 | 1.14 | UGU(C) | 33 | 1.02 |
| UUC(F) | 50 | 0.91 | UCC(S) | 91 | 1.39 | UAC(Y) | 88 | 0.86 | UGC(C) | 32 | 0.98 |
| UUA(L) | 107 | 1.40 | UCA(S) | 82 | 1.25 | UAA(*) | 106 | 1.33 | UGA(*) | 53 | 0.67 |
| UUG(L) | 57 | 0.75 | UCG(S) | 29 | 0.44 | UAG(*) | 80 | 1.00 | UGG(W) | 37 | 1.00 |
| CUU(L) | 74 | 0.97 | CCU(P) | 105 | 1.34 | CAU(H) | 89 | 1.11 | CGU(R) | 28 | 0.74 |
| CUC(L) | 62 | 0.81 | CCC(P) | 76 | 0.97 | CAC(H) | 72 | 0.89 | CGC(R) | 27 | 0.71 |
| CUA(L) | 117 | 1.53 | CCA(P) | 109 | 1.39 | CAA(Q) | 96 | 1.26 | CGA(R) | 28 | 0.74 |
| CUG(L) | 41 | 0.54 | CCG(P) | 24 | 0.31 | CAG(Q) | 56 | 0.74 | CGG(R) | 34 | 0.90 |
| AUU(I) | 117 | 1.29 | ACU(T) | 116 | 1.27 | AAU(N) | 116 | 1.01 | AGU(S) | 45 | 0.69 |
| AUC(I) | 81 | 0.89 | ACC(T) | 99 | 1.09 | AAC(N) | 113 | 0.99 | AGC(S) | 82 | 1.25 |
| AUA(I) | 75 | 0.82 | ACA(T) | 112 | 1.23 | AAA(K) | 81 | 1.49 | AGA(R) | 47 | 1.24 |
| AUG(M) | 48 | 1.00 | ACG(T) | 37 | 0.41 | AAG(K) | 28 | 0.51 | AGG(R) | 63 | 1.67 |
| GUU(V) | 13 | 0.71 | GCU(A) | 18 | 0.60 | GAU(D) | 33 | 0.93 | GGU(G) | 10 | 0.51 |
| GUC(V) | 10 | 0.55 | GCC(A) | 54 | 1.79 | GAC(D) | 38 | 1.07 | GGC(G) | 18 | 0.92 |
| GUA(V) | 35 | 1.92 | GCA(A) | 45 | 1.49 | GAA(E) | 42 | 1.12 | GGA(G) | 37 | 1.90 |
| GUG(V) | 15 | 0.82 | GCG(A) | 4 | 0.13 | GAG(E) | 33 | 0.88 | GGG(G) | 13 | 0.67 |
| ***C. heteronema*** | | | | | | | | | | | |
| Codon | Count | RSCU | Codon | Count | RSCU | Codon | Count | RSCU | Codon | Count | RSCU |
| UUU(F) | 92 | 1.25 | UCU(S) | 59 | 1.00 | UAU(Y) | 118 | 1.19 | UGU(C) | 33 | 0.86 |
| UUC(F) | 55 | 0.75 | UCC(S) | 72 | 1.22 | UAC(Y) | 80 | 0.81 | UGC(C) | 44 | 1.14 |
| UUA(L) | 108 | 1.41 | UCA(S) | 75 | 1.27 | UAA(*) | 111 | 1.42 | UGA(*) | 45 | 0.57 |
| UUG(L) | 61 | 0.80 | UCG(S) | 21 | 0.36 | UAG(*) | 79 | 1.01 | UGG(W) | 48 | 1.00 |
| CUU(L) | 69 | 0.90 | CCU(P) | 98 | 1.21 | CAU(H) | 79 | 1.01 | CGU(R) | 32 | 0.83 |
| CUC(L) | 59 | 0.77 | CCC(P) | 93 | 1.15 | CAC(H) | 77 | 0.99 | CGC(R) | 26 | 0.68 |
| CUA(L) | 105 | 1.38 | CCA(P) | 102 | 1.26 | CAA(Q) | 94 | 1.31 | CGA(R) | 29 | 0.75 |
| CUG(L) | 56 | 0.73 | CCG(P) | 30 | 0.37 | CAG(Q) | 49 | 0.69 | CGG(R) | 28 | 0.73 |
| AUU(I) | 124 | 1.41 | ACU(T) | 99 | 1.16 | AAU(N) | 115 | 1.04 | AGU(S) | 52 | 0.88 |
| AUC(I) | 72 | 0.82 | ACC(T) | 100 | 1.17 | AAC(N) | 106 | 0.96 | AGC(S) | 75 | 1.27 |
| AUA(I) | 68 | 0.77 | ACA(T) | 105 | 1.23 | AAA(K) | 81 | 1.56 | AGA(R) | 55 | 1.43 |
| AUG(M) | 54 | 1.00 | ACG(T) | 37 | 0.43 | AAG(K) | 23 | 0.44 | AGG(R) | 61 | 1.58 |
| GUU(V) | 18 | 0.89 | GCU(A) | 28 | 0.83 | GAU(D) | 34 | 0.99 | GGU(G) | 11 | 0.47 |
| GUC(V) | 20 | 0.99 | GCC(A) | 53 | 1.57 | GAC(D) | 35 | 1.01 | GGC(G) | 28 | 1.20 |
| GUA(V) | 24 | 1.19 | GCA(A) | 49 | 1.45 | GAA(E) | 46 | 1.28 | GGA(G) | 26 | 1.12 |
| GUG(V) | 19 | 0.94 | GCG(A) | 5 | 0.15 | GAG(E) | 26 | 0.72 | GGG(G) | 28 | 1.20 |

**Table S7** Detailed comparison of anticodon sequences present in tRNA genes across the mitogenomes of four *Cyclocheilichthys* species.

| **Genes** | ***C. repasson*** | ***C. jantochir*** | ***C. apogon*** | ***C. heteronema*** |
| --- | --- | --- | --- | --- |
| *tRNA-Phe* | GAA | GAA | GAA | GAA |
| *tRNA-Val* | TAC | TAC | TAC | TAC |
| *tRNA-Leu* | TAA | TAA | TAA | TAA |
| *tRNA-Ile* | GAT | GAT | GAT | GAT |
| *tRNA-Gln* | TTG | TTG | TTG | TTG |
| *tRNA-Met* | CAT | CAT | CAT | CAT |
| *tRNA-Trp* | TCA | TCA | TCA | TCA |
| *tRNA-Ala* | TGC | TGC | TGC | TGC |
| *tRNA-Asn* | GTT | GTT | GTT | GTT |
| *tRNA-Cys* | GCA | GCA | GCA | GCA |
| *tRNA-Tyr* | GTA | GTA | GTA | GTA |
| *tRNA-Ser* | TGA | TGA | TGA | TGA |
| *tRNA-Asp* | GTC | GTC | GTC | GTC |
| *tRNA-Lys* | TTT | TTT | TTT | TTT |
| *tRNA-Gly* | TCC | TCC | TCC | TCC |
| *tRNA-Arg* | TCG | TCG | TCG | TCG |
| *tRNA-His* | GTG | GTG | GTG | GTG |
| *tRNA-Ser* | GCT | GCT | GCT | GCT |
| *tRNA-Leu* | TAG | TAG | TAG | TAG |
| *tRNA-Glu* | TTC | TTC | TTC | TTC |
| *tRNA-Thr* | TGT | TGT | TGT | TGT |
| *tRNA-Pro* | TGG | TGG | TGG | TGG |

**Table S8** Pairwise genetic distances among three *Cyclocheilichthys* species based on K2P model estimated from partial *COI* gene sequences.

| **Accession No._Valid Species Name** | **Pairwise genetic distance (%)** | | | | | | | | | | | |
| --- | --- | --- | --- | --- | --- | --- | --- | --- | --- | --- | --- | --- |
| PX022682_*Cyclocheilichthys repasson* |  |  |  |  |  |  |  |  |  |  |  |  |
| PP937077_*Cyclocheilichthys repasson* | 0 |  |  |  |  |  |  |  |  |  |  |  |
| JQ346165_*Cyclocheilichthys repasson* | 1.06 | 1.06 |  |  |  |  |  |  |  |  |  |  |
| KP712065_*Cyclocheilichthys apogon* | 9.23 | 9.23 | 9.23 |  |  |  |  |  |  |  |  |  |
| OR288570_*Cyclocheilichthys apogon* | 9.44 | 9.44 | 9.86 | 0.53 |  |  |  |  |  |  |  |  |
| MW591042_*Cyclocheilichthys apogon* | 9.65 | 9.65 | 10.10 | 0.70 | 0.17 |  |  |  |  |  |  |  |
| MW591043_*Cyclocheilichthys apogon* | 9.65 | 9.65 | 10.10 | 0.70 | 0.17 | 0 |  |  |  |  |  |  |
| MW591044_*Cyclocheilichthys apogon* | 9.65 | 9.65 | 10.10 | 0.70 | 0.17 | 0 | 0 |  |  |  |  |  |
| MW591045_*Cyclocheilichthys apogon* | 9.65 | 9.65 | 10.10 | 0.70 | 0.17 | 0 | 0 | 0 |  |  |  |  |
| HM536907_*Cyclocheilichthys janthochir* | 9.66 | 9.66 | 10.10 | 4.55 | 4.36 | 4.55 | 4.55 | 4.55 | 4.55 |  |  |  |
| JF915567_*Cyclocheilichthys janthochir* | 9.66 | 9.66 | 10.10 | 4.55 | 4.36 | 4.55 | 4.55 | 4.55 | 4.55 | 0 |  |  |
| MN342372_*Cyclocheilichthys janthochir* | 9.66 | 9.66 | 10.10 | 4.55 | 4.36 | 4.55 | 4.55 | 4.55 | 4.55 | 0 | 0 |  |
| MN342373_*Cyclocheilichthys janthochir* | 9.66 | 9.66 | 10.10 | 4.55 | 4.36 | 4.55 | 4.55 | 4.55 | 4.55 | 0 | 0 | 0 |
